# BCG-induced trained immunity enhances acellular pertussis vaccination responses in an explorative randomized clinical trial

**DOI:** 10.1038/s41541-022-00438-4

**Published:** 2022-02-17

**Authors:** Joshua Gillard, Bastiaan A. Blok, Daniel R. Garza, Prashanna Balaji Venkatasubramanian, Elles Simonetti, Marc J. Eleveld, Guy A. M. Berbers, Pieter G. M. van Gageldonk, Irma Joosten, Ronald de Groot, L. Charlotte J. de Bree, Reinout van Crevel, Marien I. de Jonge, Martijn A. Huynen, Mihai G. Netea, Dimitri A. Diavatopoulos

**Affiliations:** 1 Section Pediatric Infectious Diseases, Laboratory of Medical Immunology, Radboud Institute for Molecular Life Sciences, Radboud University Medical Center, 6500 HB Nijmegen, The Netherlands; 2 Radboud Center for Infectious Diseases, Radboud University Medical Center, 6500 HB Nijmegen, The Netherlands; 3 Center for Molecular and Biomolecular Informatics, Radboud University Medical Center, 6526 GA Nijmegen, The Netherlands; 4 Laboratory for Medical Immunology, Radboud University Medical Center, 6500 HB Nijmegen, the Netherlands; 5 Department of Internal Medicine and Radboud Center for Infectious Diseases (RCI), Radboud University Medical Center, 6526 GA Nijmegen, The Netherlands; 6 Research Center for Vitamins and Vaccines, Bandim Health Project, Statens Serum Institut, DK-2300 Copenhagen, Denmark; 7 Odense Patient Data Explorative Network, University of Southern Denmark/Odense University Hospital, DK-5000 Odense, Denmark; 8 Centre for Infectious Disease Control, National Institute of Public Health and the Environment, 3720 BA Bilthoven, The Netherlands; 9 Department for Genomics & Immunoregulation, Life and Medical Sciences Institute (LIMES), University of Bonn, Bonn, Germany

**Keywords:** Vaccines, Infectious diseases

## Abstract

Acellular pertussis (aP) booster vaccines are central to pertussis immunization programs, although their effectiveness varies. The Bacille Calmette-Guérin (BCG) vaccine is a prototype inducer of trained immunity, which enhances immune responses to subsequent infections or vaccinations. While previous clinical studies have demonstrated that trained immunity can protect against heterologous infections, its effect on aP vaccines in humans is unknown. We conducted a clinical study in order to determine the immunological effects of trained immunity on pertussis vaccination. Healthy female volunteers were randomly assigned to either receive BCG followed by a booster dose of tetanus-diphteria-pertussis inactivated polio vaccine (Tdap-IPV) 3 months later (BCG-trained), BCG + Tdap-IPV concurrently, or Tdap-IPV followed by BCG 3 months later. Primary outcomes were pertussis-specific humoral, T- and B-cell responses and were quantified at baseline of Tdap-IPV vaccination and 2 weeks thereafter. As a secondary outcome in the BCG-trained cohort, ex vivo leukocyte responses were measured in response to unrelated stimuli before and after BCG vaccination. BCG vaccination 3 months prior to, but not concurrent with, Tdap-IPV improves pertussis-specific Th1-cell and humoral responses, and also increases total memory B cell responses. These responses were correlated with enhanced IL-6 and IL-1β production at the baseline of Tdap-IPV vaccination in the BCG-trained cohort. Our study demonstrates that prior BCG vaccination potentiates immune responses to pertussis vaccines and that biomarkers of trained immunity are the most reliable correlates of those responses.

## Introduction

Pertussis is a highly transmissible acute respiratory disease caused by the bacterium *Bordetella pertussi*s and has re-emerged a serious public health issue despite high vaccine coverage^1^. For years, the disease was controlled in industrialized countries through vaccination with whole-cell pertussis vaccines (wPs) that were implemented in the 1940s-1950s. These vaccines were highly effective and induced long-term protection. However, high reactogenicity and safety concerns led to their replacement in the 1990s–2000s by acellular pertussis vaccines (aPs). aP booster vaccines, including combination vaccines that contain tetanus and diphteria toxoid (Tdap) either with or without inactivated poliovirus (IPV) (Tdap-IPV), were introduced shortly thereafter. aP vaccines are now widely used to boost immunity in pre-school children, adolescents, and adults. Despite these measures and their improved safety profile, multiple studies have shown that aP-induced immunity remains suboptimal^2^ and wanes over time^3^. Furthermore, the duration of protection after repetitive aP doses progressively shortens^4–6^. The growing need for new and improved vaccination strategies has led to the exploration of new adjuvants that target innate immunity and enhance specific immunity to aPs^7–9^.

The Bacille Calmette-Guérin (BCG) vaccine provides protection against mycobacterial infections such as tuberculosis^10^, as well as protection against secondary infections with unrelated pathogens in mouse models^1^. In humans, these non-specific beneficial effects have been linked to reduced infant mortality^11,12^ and have been validated in randomized clinical trials^10,13^. BCG induces long-term changes in innate immune cells that enhances their responsiveness to stimulation with unrelated pathogens. This process, called trained immunity, involves the epigenetic reprogramming of monocytes and persists for up to a year after BCG vaccination^14^. The clinical relevance of trained immunity has been demonstrated in humans through studies that use BCG as the prototype for the induction of trained immunity and vaccination or experimental human infection as the secondary immune perturbation^15–17^. There is accumulating evidence that BCG vaccination modulates adaptive immune responses to vaccines. Among eight studies that were evaluated in a recent review, five showed enhanced vaccine-specific humoral responses^18^.

Efforts to delineate the interacting effects of BCG and pertussis vaccines suggest that BCG-induced trained immunity may have a positive effect on the immune response to pertussis. In mice, prior vaccination with BCG enhanced Th1 and humoral immune responses following aP, but not wP vaccination^19^. In humans, one population-scale study showed that co-administration of BCG with pertussis vaccination reduced mortality in infants^20^ compared to receiving the vaccines sequentially. To the best of our knowledge, no study to date has investigated in a randomized controlled study whether trained immunity modulates immune responses to pertussis vaccines or has compared the timing of administration of BCG vaccination relative to a second vaccination or challenge. Here, we tested whether BCG vaccination impacts the adaptive immune response to aP vaccination, and whether trained immunity is associated with those responses. We show that BCG-induced trained immunity enhances aP-specific antibody, aP-specific Th1 cell responses and total memory B cell responses 2 weeks post Tdap-IPV immunization. These increases were positively correlated with increases in trained immunity biomarkers, including IL-1β and IL-6. Our findings highlight a role for trained immunity induced by BCG vaccination in potentiating immunity to pertussis vaccines.

## Results

### Study design

**Fig. 1:**
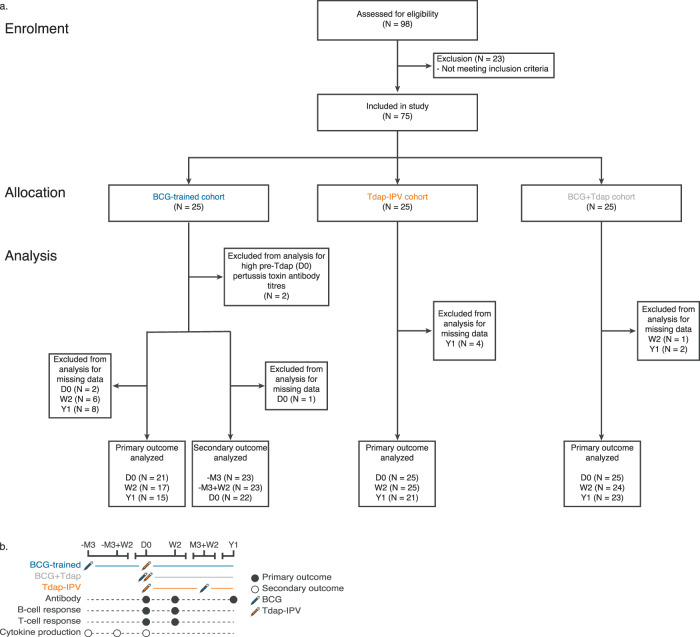
Flow diagram and study procedures. **a** Flow diagram of subjects who were enrolled in the study and analyzed. Seventy-five female subjects were randomly assigned to one of three study cohorts. **b** Overview of the study design and measurements. Antibody, B-cell and T-cell responses were measured across all three cohorts (primary outcomes, black circles). Ex vivo peripheral blood mononuclear cell cytokine production in response to heterologous stimulation with heat-killed microbes or microbial ligands was measured in the BCG-trained cohort (secondary outcomes, white circles). Timestamps are defined relative to the baseline of Tdap-IPV vaccination (-M3 = 3 months prior, -M3 + W2 = 3 months prior plus 2 weeks, D0 baseline of Tdap-IPV or concurrent Tdap-IPV and BCG vaccinations, W2 2 weeks thereafter, M3 + W2 3 months plus 2 weeks thereafter, Y1 1 year thereafter).

Seventy-five healthy female volunteers were randomized to one of three cohorts to receive BCG and Tdap-IPV vaccines in different orders^21^. We chose to enroll only women since gender-specific effects of pertussis vaccines have been reported^22^. The median age of the volunteers was 23, with no significant difference in age between cohorts (Kruskal–Wallis test; *P* = 0.6), as previously reported^21^. Two subjects were excluded from analysis for having a high anti-pertussis toxin IgG concentration prior to Tdap-IPV vaccination (>100 IU/ml), indicative of recent infection with pertussis^23,24^ (Fig. 1a). In order to identify changes in the Tdap-IPV vaccine response that are associated with trained immunity, the “BCG-trained” cohort received BCG 3 months prior to Tdap-IPV. We also included two control cohorts to account for the timing of the BCG vaccination. The “BCG + Tdap” cohort received both vaccinations in opposite limbs at the same time, and the “Tdap-IPV” cohort received Tdap-IPV first and BCG 3 months later (Fig. 1b). Blood was collected at several time points before and after the vaccinations. Timestamps are defined relative to the baseline of Tdap-IPV vaccination (-M3 = 3 months prior, -M3 + W2 = 3 months prior plus 2 weeks, D0 = baseline of Tdap-IPV or concurrent BCG and Tdap-IPV vaccinations, W2 = 2 weeks thereafter, M3 + W2 = 3 months plus 2 weeks thereafter, Y1 = 1 year thereafter). Primary outcomes were specified as increases in adaptive immune responses 2 weeks or 1 year after Tdap-IPV vaccination. In total, we assessed 31 immunological outcomes across three vaccination cohorts. We used a bead-based multiplex immunoassay to measure antibody concentrations of IgG against pertussis antigens pertussis toxin (PT), filamentous haemagglutinin (FHA), pertactin (PRN), as well as diphtheria toxoid (DIPH) and tetanus toxin (TET)^25^. Total B-cell and PRN-specific B-cells were measured directly in whole blood by flow cytometry using fluorescently labeled PRN (Supplementary Fig. 1). Th1 and Th17 T-cell responses against pertussis antigens were measured by stimulating peripheral blood mononuclear cells (PBMCs) with either PRN, PT, or FHA for 7 days and measuring secreted IFNγ, IL-22, and IL-17 by ELISA^26^.

BCG vaccination enhances immune cell cytokine responses to unrelated stimuli, a phenomenon that has been explained by two different biological mechanisms. The first process is called heterologous immunity and depends on non-specific activation of T cells^27,28^. The second process, called trained immunity, results in increased pro-inflammatory cytokine production by innate immune cells^14^. We defined secondary outcomes for the BCG-trained cohort as changes in cytokine production of PBMCs following stimulation with heat-killed *Candida albicans* (C_alb), *Staphylococcus aureus* (S_aur), *Bordetella pertussis* (Bp), or lipopolysaccharide (LPS). As in previous studies^16,21^, we quantified Th1/17 cytokines IFNγ, IL-22, or IL-17 after 7 days as a readout for heterologous immunity, or monocyte-derived cytokines IL-10, IL-6, IL-1β, or TNF after 24 h as a readout for trained immunity (Fig. 1b). Cytokine production in response to sonicated *Mycobacterium tuberculosis* (Mtb) was measured to quantify anti-mycobacterial immunity.

### Prior BCG vaccination enhances Tdap-IPV-induced adaptive immune responses

**Fig. 2:**
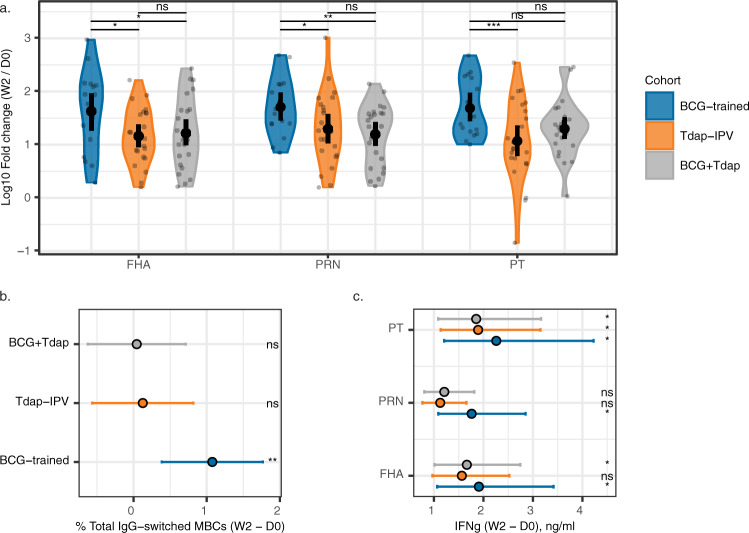
Prior BCG vaccination enhances pertussis-specific antibody, IFNg, and total memory B cell responses 2 weeks following Tdap-IPV vaccination. **a** Violin plots showing log10 fold change (W2/D0) of pertussis toxin (PT), pertactin (PRN), and filamentous haemagglutinin (FHA) -specific IgG responses, data are N = 16–25 per cohort. **b** Differences in total IgG-switched memory B cell (MBC) responses (W2 – D0), data are *N* = 15–16 per cohort, and **c** differences in IFNg produced in response to PT, PRN, or FHA stimulation of peripheral blood mononuclear cells (W2–D0), data are *N* = 16–25 per cohort. *P*-values, significance, and estimates were calculated with a linear mixed model fitting each primary outcome with time, cohort, and their interaction as main effects. Sample means are plotted with 95% confidence intervals (solid black point and line), *p < 0.05; **p < 0.01; ***p < 0.001; ns not significant.

In order to quantify the effects of trained immunity on Tdap-IPV vaccination, for each primary endpoint we constructed a linear mixed-effects model with sample time, cohort membership, as well as the interaction of time and cohort as covariates. We examined two contrasts: (i) differences compared to baseline (D0) within each cohort (post-vaccination effects, Supplementary Table 1), and (ii) between-cohort differences in the magnitude of those changes (differential effects, Supplementary Table 2). All cohorts mounted significant increases in antibody responses to the five antigens following Tdap-IPV immunization. Antibody concentrations remained higher than baseline up to a year post-vaccination (Y1), with the exception of TET, which had waned in the BCG-trained and BCG + Tdap cohorts (Supplementary Fig. 2a). All cohorts had significantly increased numbers of circulating PRN + B cells 2 weeks post immunization, which were mainly memory B cells (MBCs) (Supplementary Fig. 3a). We did not observe differences between cohorts (Supplementary Table 2 and Supplementary Fig. 3b). Compared to the Tdap-IPV cohort, the BCG-trained cohort displayed enhanced baseline-normalized log10 fold changes (W2/D0) for IgG responses for PT, FHA, and PRN. A similar pattern was observed when comparing these responses to the BCG + Tdap cohort (Fig. 2a). We also observed elevated pertussis-specific IgG concentrations in the BCG-trained cohort 2 weeks post-vaccination (Supplementary Fig. 2b), although the difference in the absolute values was less pronounced than the difference in the increases that are relative to the baseline at D0. Furthermore, subjects in the BCG-trained cohort displayed significantly increased total MBC responses, including IgG class-switched MBCs (Fig. 2b and Supplementary Fig. 4). Subjects in the BCG-trained cohort also displayed elevated IFNγ secretion in response to stimulation with all three pertussis antigens. Conversely, significant increases were observed for a single pertussis antigen in the Tdap-IPV cohort (PT) and two pertussis antigens in the BCG + Tdap cohort (PT and FHA, Fig. 2c and Supplementary Fig. 5). We also observed a trend where increases in IFNg production were higher in the BCG-trained cohort than in the other cohorts, although this did not reach statistical significance (Supplementary Table 2 and Supplementary Fig. 5b). Pertussis-specific IL-22 and IL-17 responses were weak for all cohorts (Supplementary Table 1). Altogether, these findings point to enhanced pertussis-specific antibody responses, enhanced total IgG-switched MBC responses, and a broader pertussis-specific IFNγ responses in the BCG-trained cohort compared to the other two cohorts.

**Fig. 3:**
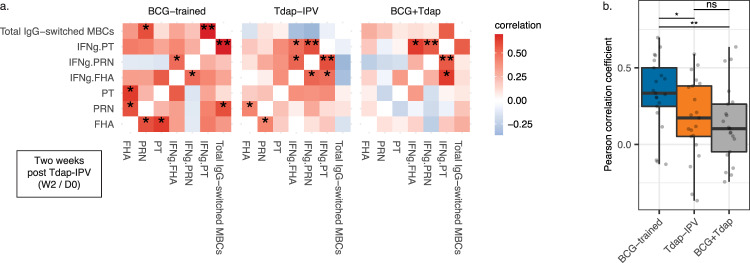
Pertussis-specific antibody, IFNg, and total memory B cell responses are highly intercorrelated in the BCG-trained cohort following Tdap-IPV vaccination. **a** Pearson correlation heatmap of increases in pertussis toxin (PT), pertactin (PRN), filamentous haemagglutinin (FHA) IgG, IFNg production following peripheral blood mononuclear cell stimulation with pertussis antigens (IFNg.PT, IFNg.PRN, IFNg.FHA), and total IgG-switched memory B cells (MBCs) 2 weeks post Tdap-IPV vaccination. The statistical significance of the correlation is shown, *p < 0.05; **p < 0.01. **b** Pearson correlation coefficients were extracted from **a** and plotted per cohort. Data are represented as a box-and-whisker plots, with bounds from 25th to 75th percentile, median line, and whiskers, which extend to the largest or smallest value no further than 1.5 × the inter-quartile range (distance between the first and third quartiles). Data were compared using the Wilcoxon signed-rank test. Data are from *N* = 16–25 subjects per cohort. *p < 0.05; **p < 0.01; ns not significant.

We explored the relationships between the increases in primary outcomes that were enhanced in the BCG-trained cohort. For this exploratory analysis, correlations between antibodies, B-cells, and cytokines were calculated for each cohort. The BCG-trained cohort displayed many significant and quantitatively stronger positive correlations between these responses, while the Tdap-IPV and BCG + Tdap cohorts displayed weaker or inverse correlations, with the strongest pattern corresponding to changes in IFNγ responses (Fig. 3a). Correlation coefficients between responses in the BCG-trained were significantly greater than those in the control cohorts, altogether pointing towards the induction of a more highly coordinated pertussis-specific adaptive immune response in the BCG-trained cohort (Fig. 3b).

### IL-1β and IL-6 cytokine production is associated with pertussis-specific adaptive immune responses

**Fig. 4:**
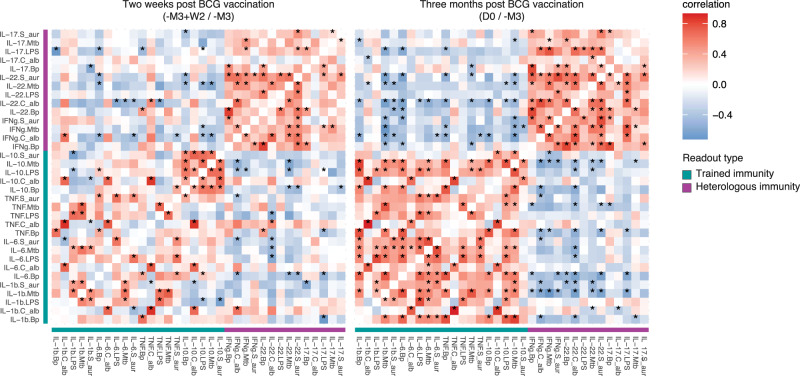
BCG vaccination induces coordinated cytokine production in response to ex vivo stimulation. Within the BCG-trained cohort, log10 fold changes were calculated at 2 weeks (-M3 + W2/-M3) and 3 months post BCG (D0/-M3) for the indicated cytokine:stimulation combinations. Pearson correlations and associated significance between these changes are shown. Cytokines are labeled as readouts for heterologous immunity (purple) or trained immunity (turquoise). Data are *N* = 23 (left) and *N* = 22 (right), *p < 0.05. LPS lipopolysaccharide, Bp *Bordetella pertussis*, C_alb *Candida albicans*, Mtb *Mycobacterium tuberculosis*, S_aur *Staphylococcus aureus*.

The BCG-trained cohort displayed enhanced total IgG-switched MBC and pertussis-specific IFNγ responses. Furthermore, compared to the Tdap-IPV and BCG + Tdap cohorts, the BCG-trained cohort also displayed higher pertussis antibody responses. Since BCG vaccination in humans can increase cytokine production of circulating immune cells in response to heterologous stimulation, weeks to months after vaccination^27^, we evaluated whether these BCG-induced cytokine responses were associated with changes in pertussis-specific immune responses following Tdap-IPV vaccination. Two weeks and 3 months after BCG vaccination, there was a significantly enhanced anti-mycobacterial response characterized by increased IL-22 and IFNγ production compared to BCG vaccination baseline (Supplementary Fig. 6a, b). Overall, the BCG-trained cohort did not show increased production of heterologous immunity or trained immunity-associated cytokines, as previously observed (Supplementary Figs. 6 and 8)^21^. However, since the induction of cytokine production varied between individuals, we calculated changes between 2 weeks post BCG and 3 months post BCG compared to BCG baseline (Supplementary Figs. 7 and 9). Pearson correlations were calculated to determine the strength of the association between these measurements at each timepoint. Three months after BCG vaccination, i.e. at the Tdap-IPV baseline, cytokine responses to in vitro stimulation were highly correlated. Heterologous immunity-associated cytokine responses were negatively correlated with trained immunity-associated cytokine responses, and amongst those, correlations between IL-6 and IL-1β were strongest (Fig. 4), as has been previously described^16^. Correlation patterns 2 weeks post BCG vaccination were similar for heterologous immunity-associated cytokines but correlations between trained immunity-associated cytokines were much weaker, with the exception of IL-10 responses. Taken together, these results point to coordinated innate immune activation 3 months, but not 2 weeks post BCG-immunization in the BCG-trained cohort.

**Fig. 5:**
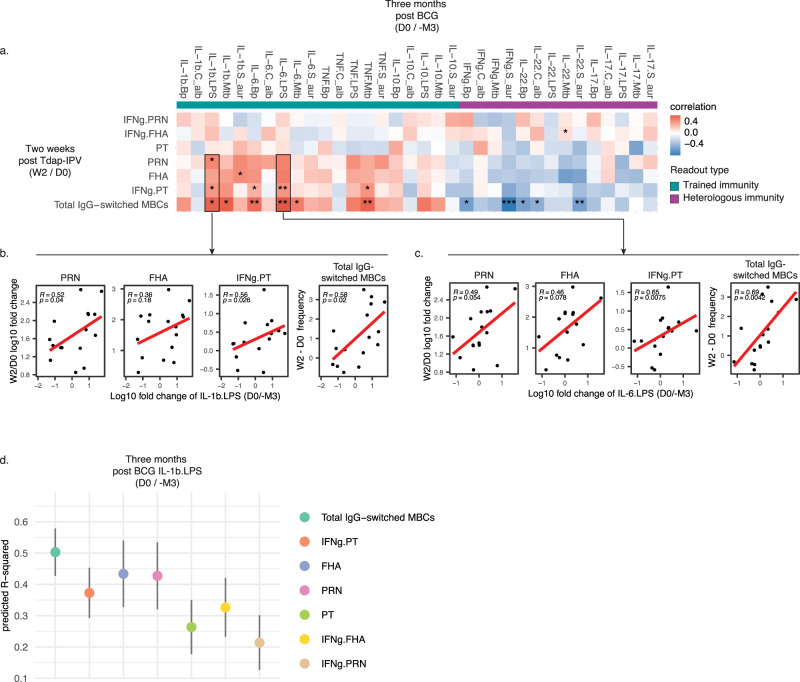
Increases in IL-1b and IL-6 following BCG vaccination are associated with increases in pertussis-specific antibody, IFNg, and total memory B cell responses following Tdap-IPV. Within the BCG-trained cohort, increases for the indicated cytokine:stimulation combinations were calculated at 3 months post BCG (D0/-M3). **a** Pearson correlations between the cytokine variables on the x-axis and primary outcome responses 2 weeks post Tdap-IPV vaccination on the y-axis: Log10 fold change (W2 /D0) pertussis toxin (PT), pertactin (PRN), filamentous haemagglutinin (FHA) IgG, pertussis-specific IFNg responses (IFNg.PT, IFNg.PRN, IFNg.FHA), and total IgG-switched memory B cells (MBCs, W2 – D0). *p < 0.05; **p < 0.01; ***p < 0.001. **b** scatterplots displaying the Pearson correlation (R) between primary outcomes PRN, FHA, IFNg.PT, and total IgG-switched MBCs and IL-1b cytokine production in response to lipopolysaccharide (LPS) stimulation (IL-1b.LPS) of peripheral blood mononuclear cells measured 3 months post-BCG (D0/-M3), **c** similar to **b**, scatterplots displaying the Pearson correlation between primary outcomes and IL-6 cytokine production in response to LPS (IL-6.LPS). *P*-values for the correlation are indicated in each plot for both **b** and **c**, as well as a linear regression line in red. **d** IL-1b.LPS was selected based on a strong correlation with multiple primary outcomes. Linear models were constructed for each indicated primary outcome and predicted R-squared was calculated following 10 times repeated, 4-fold cross-validation. The mean predicted R-squared values are shown with 95% confidence intervals. Data are *N* = 16 subjects in the BCG-trained cohort.

Next, we explored whether variations in the cytokine responses of the BCG-trained cohort were associated with changes in Tdap-IPV adaptive immune endpoints. We examined changes in cytokine production 2 weeks and 3 months post BCG vaccination. We found that increases in heterologous immunity-associated cytokines tended to be weakly, and negatively, correlated with the primary endpoints at both 2 weeks (Supplementary Fig. 10) and 3 months post BCG-vaccination (Fig. 5a). By contrast, increases in trained immunity-associated cytokines 3 months after BCG vaccination, particularly IL-1β/IL-6 in response to LPS stimulation, were significantly positively correlated with PRN and FHA antibody responses, IFNγ production in response to PT stimulation, and total IgG-switched MBC responses (Fig. 5b, c). In line with this, we did not observe changes in the relative abundance of peripheral blood cell numbers (Supplementary Fig. 11a), nor were changes in cellular abundance 3 months post-BCG vaccination positively correlated with pertussis endpoints (Supplementary Fig. 11b). Increases in IL-1β production in response to LPS stimulation (IL1b.LPS) 3 months post BCG vaccination showed a strong association with multiple Tdap-IPV responses and was selected to determine how much of the variation of each response they could explain. The total variance explained (predicted Rsq) for each outcome was calculated and changes in IL-1β could explain up to 60% of the response variation (Fig. 5d). Overall these results highlight that trained immunity-associated cytokine responses 3 months after BCG vaccination are correlated with increases in pertussis-specific antibody responses and IFNγ production, as well as total IgG-switched MBC responses.

## Discussion

Induction of trained immunity in humans occurs weeks to months after BCG vaccination^14^ and can affect the response to subsequent immunization in humans^16,17^. Here, we show that trained immunity that is induced by BCG vaccination 3 months prior to, but not concurrent with, a booster dose of Tdap-IPV, augments pertussis IgG responses and modulates pertussis-specific cellular responses. Several studies have investigated the interacting effects of BCG and other vaccines, with most of these studies reporting a positive effect of BCG on pertussis immune responses^20,21,29,30^. However, to the best of our knowledge no study to date has investigated the timing of BCG vaccination on the response to pertussis or studied which features of the BCG-induced trained immunity response are correlated with enhanced vaccine responsiveness.

Subjects in all three cohorts mounted anti-pertussis humoral and PRN + B cell responses 2 weeks post vaccination with Tdap-IPV. Although PRN antibody responses were much higher in the BCG-trained cohort, we did not observe a similar pattern in PRN-specific B cell responses. This could possibly be explained by a faster induction of antibodies in the BCG-trained cohort, which accumulates over time due to their long half-life. However, since antibody concentrations were not measured before 2 weeks post-vaccination, it was not possible to estimate differences in the kinetics between cohorts. Alongside increased PRN antibody responses, we also found that FHA and PT antibody responses were higher in the BCG-trained cohort. We were unfortunately unable to measure PT- and FHA-specific B cell responses due to high non-specific binding of FHA and PT to B cells. Still, it is promising that total IgG MBC responses were increased in the BCG-trained cohort and that these increases were correlated with increases in antibody responses. We also found that IFNγ responses in response to pertussis antigen re-stimulation were enhanced in the BCG-trained cohort. Although these changes were relatively small, we observed a trend where W2–D0 changes were higher than in the other cohorts. Further studies with more subjects may help validate this pattern, which has been confirmed in a mouse study^19^. Similarly, neonatal BCG vaccination has also been shown to potentiate Th1 responses to tetanus toxoid^31^.

Increases in pertussis antibodies, total IgG MBC responses, and pertussis IFNg responses were strongly correlated with each other in the BCG-trained cohort, suggesting the coordinated activation of effector and memory functions. Furthermore, increases in pertussis antibodies, total IgG-switched MBC responses, and IFNγ in response to PT restimulation in the BCG-trained cohort were associated with innate responses, in particular IL-1β and IL-6. These results are in line with a previous human study from our group where we studied the effect of BCG on influenza vaccination^17^. The importance of the IL-1β/IL-6 pathway as a biomarker of trained immunity has also been highlighted in a model of experimental viral infection following yellow fever vaccination^16^. Overall, we did not observe correlations between particular microbial stimuli and the primary outcomes, which suggests that overall changes in cytokine response pathways and not any particular stimulation condition are linked to enhanced responsiveness to pertussis vaccination. While we expect the predominant IL-6 and IL-1β response to microbial stimulation to be derived from monocytes and dendritic cells^16^, B cell may also contribute to cytokine production^32^ and we cannot rule out their potential contribution.

A key question has been how long BCG-induced trained immunity persists post-vaccination. In vitro signatures of BCG-induced trained immunity in humans have been identified as early as 2 weeks and up to 1-year post vaccination^14^. Clinical studies in humans that have aimed to translate these findings to in vivo responsiveness have restricted the window of observation to up to 5 weeks post BCG vaccination^15–17^. We observed a weaker induction of the trained immunity-associated cytokines IL-6 and IL-1β than in previous studies^16^, as has been previously reported^21^. Batch-to-batch variations in the production of the BCG vaccine may influence the induction of trained immunity and may explain the effect that we observed in this study^33^. Furthermore, variations between BCG strains have also been observed that impact their immunogenicity and clinical efficacy^34–36^. Despite this, we nonetheless demonstrate that heterologous IL-1β and IL-6 responses 3 months after BCG vaccination, i.e. at the moment of Tdap-IPV vaccination, but not 2 weeks after BCG vaccination are associated with enhanced pertussis humoral and cellular immunity. These findings imply that trained immunity continues to develop past 2 weeks after BCG vaccination, and emphasize that the baseline functional ‘responsiveness’ of circulating innate immune cells may be critical for the development of a more potent vaccine response.

This study clearly demonstrates that prior BCG vaccination can enhance responsiveness to aP vaccine antigens. Surprisingly, we did not observe this effect for TET and DIPH. This may be explained by the fact that all adults had received a prior booster vaccination with Td-IPV but not with aP at the age of 9 years, as recommended in the national immunization program. In line with this, we found that the baseline antibody concentrations of aP antigens were highly correlated across the subjects in our study, but were not correlated with DIPH and TET, suggesting that humoral immunity to these components are not synchronized with the aP antigens (Supplementary Fig. 12). Baseline DIPH and TET antibody levels were highly intercorrelated, which is consistent with their co-administration in vaccines. Thus, variations in pre-existing immunity against different vaccine components may play a role in the capacity of BCG vaccination and trained immunity to influence the response to a Tdap-IPV.

Although our study was not designed to investigate vaccine efficacy, a major question is whether the enhanced antibody response that we observed in the BCG-trained cohort may translate into better clinical protection. There is much debate about correlates of protection against pertussis^37^. Nonetheless, PT antibodies have previously been used to correlate with clinical protection against disease (>25 IU/ml)^29^. Using this threshold, near-complete seroprotection was achieved 2 weeks post Tdap-IPV in the BCG-trained and BCG + Tdap cohorts, while a fraction of subjects in the Tdap-IPV cohort were not seroprotected (Supplementary Fig. 13). Besides antibodies, T-cell immunity and in particular Th1 and Th17 responses have also been highlighted as important for protection against pertussis in animal models^2,38^. We found that the BCG-trained cohort displayed augmented IFNγ in response to pertussis antigen re-stimulation, which is a readout for Th1 immunity, and may therefore suggest improved clinical protection against pertussis. Finally, current immunological endpoints aimed at quantifying pertussis-specific adaptive immunity, such as T-cell, B-cell, or humoral responses, have not yet been validated as immunological correlates of protection. There are several possibilities for closing this knowledge gap, for example, by conducting epidemiological studies or by the use of the recently established controlled human infection model for *B. pertussis*^39^.

A limitation of our explorative study is that the study population consisted of adults who were primed in infancy with the whole-cell DTP vaccine, which is known to influence the adaptive response to pertussis decades after priming and affects the induction of T-cell immunity in response to aP booster vaccination^40^. Thus, it will be important to determine whether our findings translate to children born in the era of routine pediatric aP vaccination. Additionally, since we included only females, it will be important to determine if differences exist with males. In this explorative study, we measured anti-pertussis immunogenicity endpoints including antibody responses, total and PRN + B cell responses, and antigen-specific T-cell responses. *P*-values were not corrected for multiple testing due to the targeted nature of the immune responses assessed. Importantly, this study was performed in volunteers of Western European (Dutch) descent, and future studies should investigate whether similar effects can be found in populations of a different genetic background. Another limitation is that only a subset of cytokines were measured. While this was appropriate for the specific hypotheses we aimed to test, a more comprehensive, unbiased profiling of cytokine and other responses may yield new insights.

In conclusion, in the present study, we report that BCG vaccination administered 3 months prior to Tdap-IPV vaccination significantly enhances anti-pertussis immunity, and that biomarkers of trained immunity are the most reliable correlates of these enhanced vaccination responses. Additional validation studies will be necessary to evaluate these findings across diverse study populations, and further immunological and genetic studies are needed to mechanistically validate the role of the IL-1 and IL-6 pathways. Altogether, the delineation of baseline innate immune function, which can be modulated by BCG, will be essential for understanding how to guide vaccine-induced adaptive immune responses towards superior immunogenicity and protection against disease.

## Methods

### Clinical trial

This single center, randomized open-label trial was performed at the Radboud University Medical Center (Nijmegen, the Netherlands) from March 2015 to July 2016^21^. The study protocol is registered at clinicaltrials.gov (NCT02771782) and was approved by the Arnhem-Nijmegen Medical Ethical Committee. Volunteers were recruited using bulletin boards and at Radboud University in Nijmegen and received moderate compensation. Volunteers who had not been previously vaccinated with BCG or recently with pertussis vaccines were included. Exclusion criteria were (i) previous BCG vaccination or recent vaccination with pertussis-containing vaccines, (ii) allergy to neomycin, polymyxin, or allergic reaction to previous vaccination with diphtheria, tetanus, pertussis, or polio vaccines, (iii) use of systemic medication other than oral contraceptive drugs, and (iv) pregnancy or history of diseases resulting from immunodeficiency. Cohort size was determined based on previous studies performed by our group in which we investigated the effects of BCG vaccination on innate immune responses^16^. Randomization across the study arms was performed with a software-based algorithm. All subjects had a history of priming with whole-cell pertussis in the first year of life. Seventy-five female volunteers were included. After written informed consent was obtained, blood was collected in ethylenediaminetetraacetic acid (EDTA) or lithium heparin (LiHep) tubes and vaccinations were administered. Complete blood counts were obtained using a Sysmex XN-450 haematology analyser. Subjects received BCG Danish 1331 (Staten Serum Institut, Copenhagen, Denmark; 0.1 ml intradermally) and Tdap-IPV (Boostrix Polio, GlaxoSmithKline; 0.5 ml intramuscularly). Sera were stored at −20 °C until analysis, and whole blood was processed for further analysis as described below. The Consolidated Standards of Reporting Trails (CONSORT) guidelines were used to prepare this manuscript (Supplementary Fig. 14).

### Serological analysis

Sera were analysed for PT-, FHA-, PRN-, TET-, and DIPH-specific IgG antibody concentrations using a fluorescent-bead-based multiplex immunoassay^25^. Antigens were covalently coupled to distinct color-coded activated carboxylated MicroPlex Microspheres (beads) (Luminex, Austin, Texas, USA). The following antigens were used for coupling: highly purified PT (Netherlands Vaccine Institute), FHA (Kaketsuken, Kumamoto, Japan), P.69 PRN expressed and purified from an *E. coli* construct^41^, diphtheria toxoid (Netherlands Vaccine Institute), and tetanus toxin (T3194, Sigma-Aldrich, Saint Louis, Missouri, USA). After a wash step in PBS, 12.5 × 10^6^ carboxylated beads/mL were activated in PBS containing 2.5 mg of 1-ethyl-3-(-3-dimethylaminopropyl)-carbodiimide hydrochloride (Thermo Fisher Scientific, Waltham, Massachusetts, USA) and 2.5 mg of N-hydroxy-sulfosuccinimide (Thermo Fisher Scientific, Waltham, Massachusetts, USA). The antigens for coupling were diluted in PBS to a concentration of 10 μg of PT, FHA, or PRN, 100 μg of DT, or 25 μg of TET per 12.5 × 10^6^ activated beads and incubated for 2 h at room temperature in the dark under constant rotation. After three wash steps, the antigen-coupled beads were stored in the dark in PBS containing 0.03% (wt/vol) sodium azide and 1% (wt/vol) bovine serum albumin at 4 °C until use. Sera diluted 1/200 and 1/4000 in PBS containing 0.1% (vol/vol) Tween 20 and 3% (wt/vol) BSA were incubated with antigen-coupled beads in a 96-well filter plate for 45 min at room temperature at 750 rpm in the dark. Reference sera in a dilution series, quality control sera, and blanks were included on each plate. The in-house reference standard for pertussis was calibrated against WHO 1st IS Pert 06/140 and serially diluted 4-fold over 6 wells (1/200 to 1/204800). The in-house reference standard for DIPH and TET was calibrated against WHO NIBSC DI-3 and TE-3 and serially diluted 4-fold over 8 wells (1/50 to 1/819200). Following incubation, wells were washed 3 times with PBS, incubated with R-phycoerythrin-labeled goat anti-human IgG antibody (Jackson Immunoresearch Laboratories, West-Grove, PA, USA) for 30 min and washed. Beads were included in PBS and median fluorescence intensity (MFI) was acquired on a Bio-Plex LX200. MFI was converted to IU/mL by interpolation from a 5-parameter logistic standard curve using Bioplex Manager 6.2 software (Bio-Rad Laboratories, Hercules, California, USA) and exported to Microsoft Excel.

### PBMC Isolation, stimulation, and cytokine detection

PBMCs were isolated by Ficoll density gradient centrifugation and resuspended at 5 × 10^6^/ml in RPMI culture medium (Roswell Park Memorial Institute medium; Invitrogen, CA, USA) supplemented with gentamycin, Glutamax (GIBCO), and pyruvate. In all, 100 μl was aliquoted per well in round-bottom 96-well plates (Corning) and stimulated with one of: RPMI (negative control), *Escherichia coli* lipopolysaccharide (LPS; 10 ng/ml, Sigma-Aldrich), sonicated *Mycobacterium tuberculosis* H37Rv (5 μg/ml), heat-killed *Staphylococcus aureus* (1 × 10^6^/ml, clinical isolate), heat-killed *Candida albicans* (1 × 10^6^/ml, UC820 strain), and heat-killed *Bordetella pertussis* (1 μg/ml, B1917 strain), PT (2 μg/ml), PRN (4 μg/ml, ReagentProteins, PFE-031), and FHA (2 μg/ml). PT and FHA were kindly provided by A. M. Buisman at the National Institute for Public Health and Environment (RIVM, Bilthoven, the Netherlands). Cells were incubated at 37 °C and 5% CO_2_ for 24 h for detection of IL-1β, IL-6, IL-10, and TNF or 7 days for IFNγ, IL-22, and IL-17. Supernatants were stored at −20 °C. Cytokines were measured in PBMC culture supernatants using enzyme-linked immunosorbent assay (ELISA) kits from R&D systems (IL-1β, TNF, IL-17, IL-22) or from Sanquin (IL-6, IL-10, IFNγ) according to the manufacturer’s instructions.

### B-cell flow cytometry

B cell staining was performed directly on whole blood with an antibody staining panel (Supplementary Table 3) including fluorescently labeled PRN for the detection of antigen-specific B cells. To label PRN-specific B cells, PRN (ReagentProteins, PFE-031) was conjugated to fluorescein (FITC Antibody labelling kit, Pierce) according to the manufacturer’s protocol (PRN-FITC). In total, 1–2 ml of freshly drawn whole blood collected in LiHep tubes was diluted in FACS buffer (PBS + 0.09% NaN3 + 0.2% bovine serum albumin, Calbiochem) and cells were pelleted and then washed twice with FACS buffer. Cells were resuspended in FACS buffer and then stained with the B cell phenotyping antibody cocktail for 15 min, fixed (FACSLysing, BD Biosciences), and after another wash with FACS buffer were analysed within 1 h of staining. Flow cytometry of PRN-antigen-specific B cells in whole blood was performed on a LSRII (BD Biosciences) flow cytometer with standardized instrument settings^42^. Flow cytometry data were analysed with Infinicyte (Version 2.0, Cytognos). Per sample, 2 × 10^6^ cells were acquired in the lymphocyte gate (Supplementary Fig. 1a). Lymphocytes were gated following the removal of doublets and debris. B cells were identified as CD45 + CD19 + and PRN-FITC specific B cells were identified. B cell subsets were identified using an adapted gating strategy^43^. We attempted to detect PT-specific B cells by flow cytometry, but we were unsuccessful due to the high background caused by non-specific staining.

### Statistics

Cytokine and antibody values were log10-transformed to account for their skewed distribution. Analysis of primary or secondary outcome responses were analysed in a mixed-effects regression model using the ‘lme4’^44^ R package. For each outcome, the formula for the model (in R notation) is:

$${{{\mathrm{Outcome}}}}\sim {{{\mathrm{Time}}}} + {{{\mathrm{Cohort}}}} + {{{\mathrm{Time:Cohort}}}} + \left( {1|{{{\mathrm{SubjectID}}}}} \right)$$

Comparisons were calculated with the ‘emmeans’^45^ R package and we examined two contrasts. First, we examined whether primary endpoints were significantly different compared to the Tdap-IPV vaccination baseline within each cohort (post-vaccination effects were defined as W2–D0 within each cohort, Supplementary Table 1). Next, we examined between-cohort differences in the magnitude of those changes (differential effects, Supplementary Table 2, defined as (W2_c1_ – D0_c1_) – (W2_c2_ – D0_c2_) where _c1_ and _c2_ refer to different cohorts for a given comparison). Changes in heterologous or trained immunity cytokines in the BCG-trained cohort were analysed in a similar fashion. The *P*-values are reported using the Kenward-Roger degrees of freedom method. Statistical parameters are reported directly in the figures and figure legends. Due to the explorative study design and the targeted immune responses assessed, no multiple testing correction was performed. Pairwise correlations were performed with log10 fold change values over baseline. The estimation of variance explained by cytokine measurements on primary outcome variables was performed using the R package ‘caret’^46^. In order to account for potential overfitting, 10-times repeated, 4-fold cross validation was performed. We analysed seroprotective antibody titers against PT, TET, or DIPH with a Fisher exact test. Associations between seroprotection status and cohort were determined by examining residuals of a chi-square test. Threshold values for seroprotection to PT were 25 IU/ml^18^ and 0.1 IU/ml for DIPH and TET^37^).

### Reporting summary

Further information on research design is available in the Nature Research Reporting Summary linked to this article.

## Supplementary information

Combined supplementary materials, figures and tables

REPORTING SUMMARY

## Data Availability

The data that support the findings of this study are available from the corresponding author upon reasonable request.

## Code availability

The R-code that supports the findings of this study is available from the corresponding author upon reasonable request.
