# Supplementary material for: BCG-induced trained immunity enhances acellular pertussis vaccination responses in an explorative randomized clinical trial
Source: NPJ Vaccines. 2022 Feb 17;7:21. doi: 10.1038/s41541-022-00438-4 (PMC8854388; doi:10.1038/s41541-022-00438-4)
Supplement: Supplementary file 1 — Combined supplementary materials, figures and tables [file 41541_2022_438_MOESM1_ESM.pdf]

BCG-induced trained immunity enhances acellular pertussis vaccination responses in an explorative randomized clinical trial

Supplementary Information

Joshua Gillard[1,2,7,8], Bastiaan A. Blok[4,5,6], Daniel Garza[7], Balaji Venkatasubramanian[7], Elles Simonetti[1,8], Marc J. Eleveld[1,8], Guy Berbers[3], Pieter G. M. van Gageldonk[3], Irma Joosten[8], Ronald de Groot[1,8], L. Charlotte J. de Bree[4], Reinout van Crevel[4], Marien I. de Jonge[1,2,8], Martijn A. Huynen[7], Mihai G. Netea[4,9], and Dimitri A. Diavatopoulos[1,2,8*]

[1]Section Pediatric Infectious Diseases, Laboratory of Medical Immunology, Radboud Institute for Molecular Life Sciences, Radboud University Medical Center, 6500 HB, Nijmegen, The Netherlands

[2]Radboud Center for Infectious Diseases, Radboud University Medical Center, 6500 HB, Nijmegen, The Netherlands

[3]Centre for Infectious Disease Control, National Institute of Public Health and the Environment, 3720 BA, Bilthoven, The Netherlands

[4]Department of Internal Medicine and Radboud Center for Infectious Diseases (RCI), Radboud University Medical Center, 6526 GA Nijmegen, The Netherlands

[5]Research Center for Vitamins and Vaccines, Bandim Health Project, Statens Serum Institut, DK-2300 Copenhagen, Denmark

[6]Odense Patient Data Explorative Network, University of Southern Denmark/Odense University Hospital, DK-5000, Odense, Denmark

[7]Center for Molecular and Biomolecular Informatics, Radboud University Medical Center, 6526 GA Nijmegen, The Netherlands

[8]Laboratory for Medical Immunology, Radboud University Medical Center, 6500 HB, Nijmegen, the Netherlands

[9]Department for Genomics & Immunoregulation, Life and Medical Sciences Institute (LIMES), University of Bonn, Germany

*Corresponding author: Dr. Dimitri Diavatopoulos

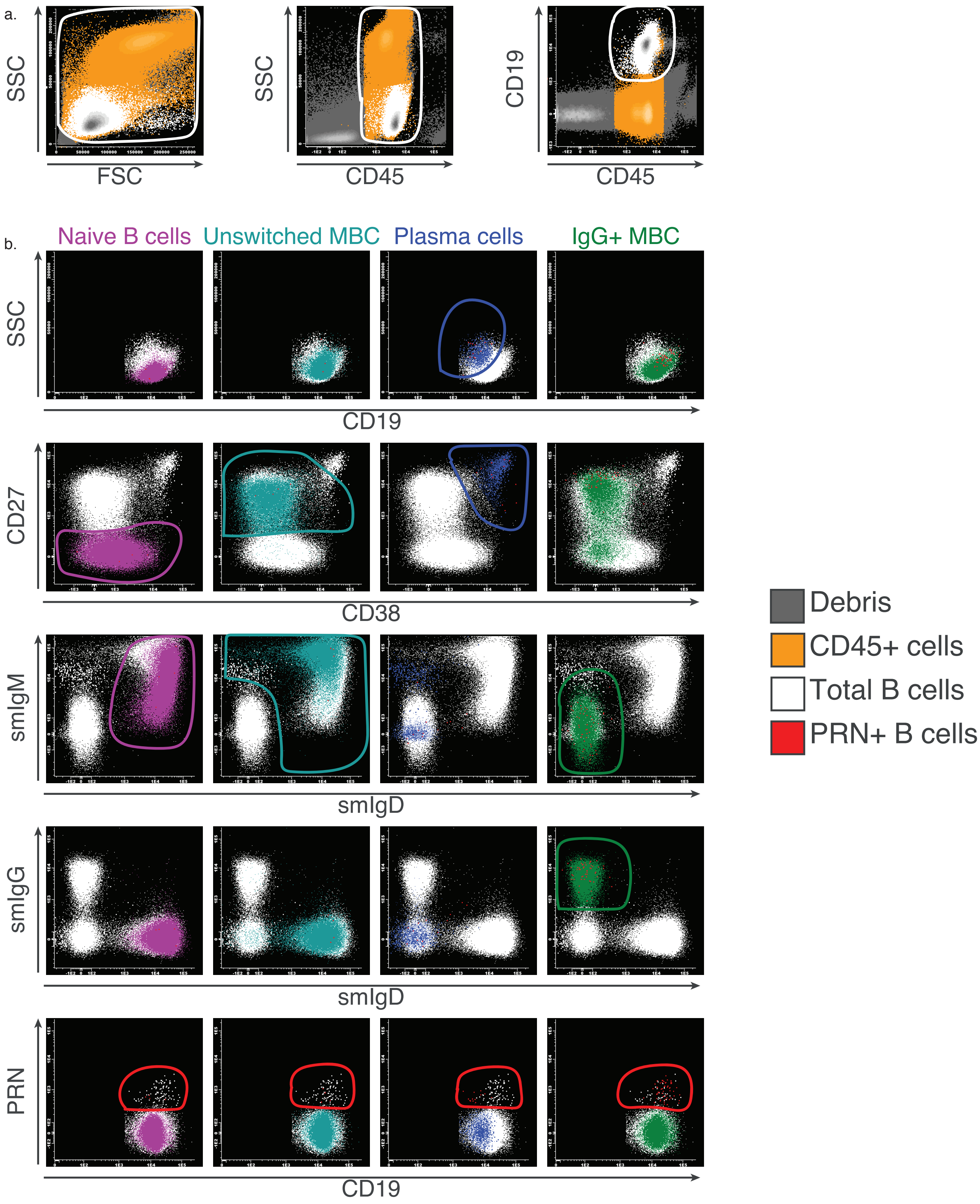

Supplementary Figure 1. Analysis strategy for the identification of PRN-specific B cell subsets and general B cell populations. a. Bivariate dot plots for identification of total B cells among CD45+ cells. Populations were classified according to their expression of CD45, CD19, as well as forward- and side-scatter characteristics (FSC and SSC). **b.** Bivariate dot plots for the identification of B cell subpopulations, including pertactin-positive (PRN+) cells. One representative sample is shown, taken from a subject two weeks post Tdap-IPV vaccination. Naïve, unswitched memory B cells (MBCs), plasma cells, and IgG+ class-switched MBCs are shown. PRN+ B cells are shown in red, and total B cells not part of the population in colour are shown in white. Cell populations were classified according to their expression of CD27, CD38, CD19, and surface membrane (sm) expression of IgH isotypes (smIgM, smIgD, and smIgG). SSC is also shown. PRN+ cells were identified as FITC+ B cells due to binding of FITC-labelled PRN.

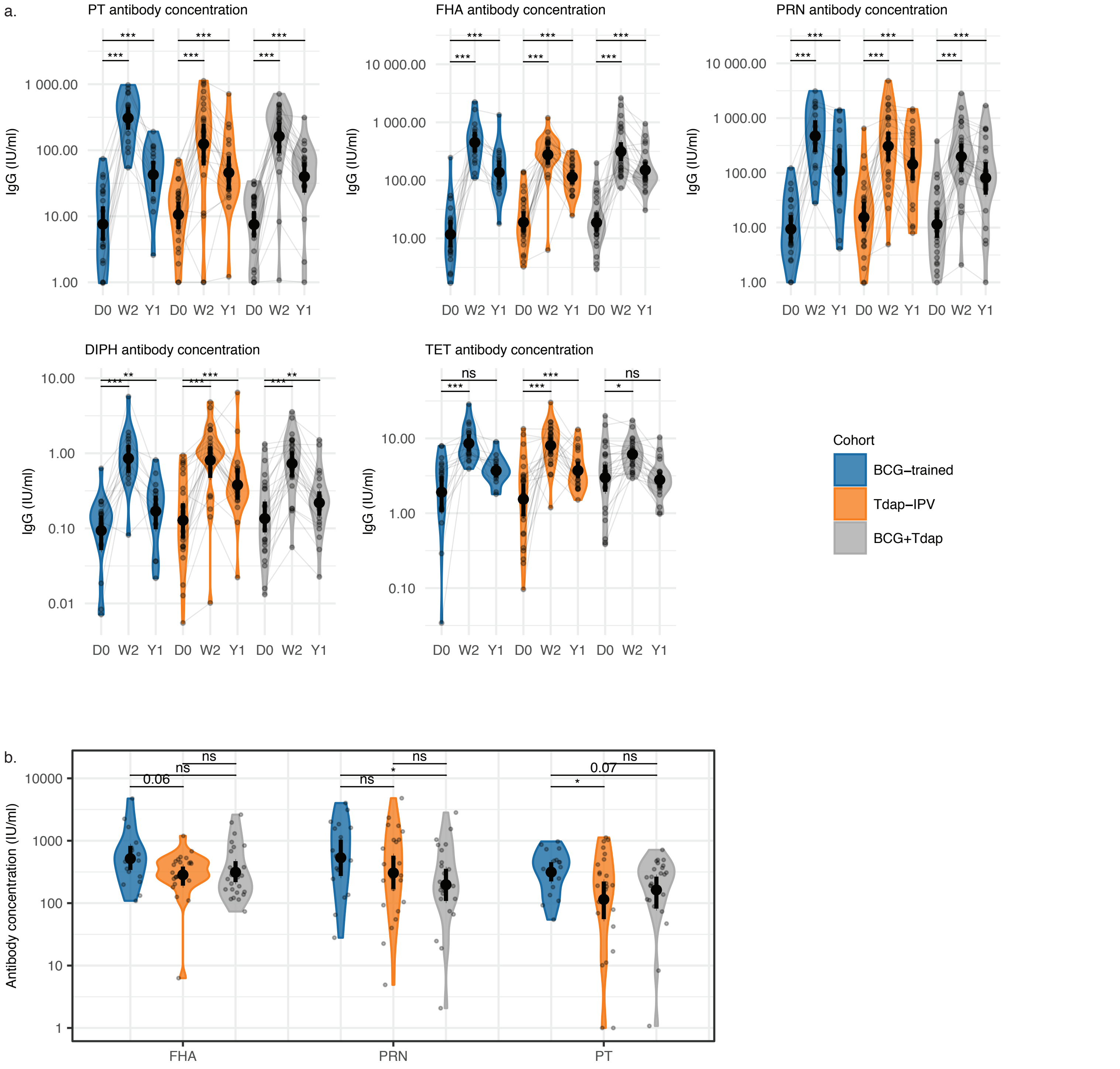

Supplementary Figure 2. Serum antibody concentrations to pertussis antigens, tetanus toxin and diphtheria toxoid following Tdap-IPV vaccination. a. Violin plots are shown for IgG concentrations at baseline of Tdap-IPV vaccination (D0), two weeks (W2), and one year (Y1) post Tdap-IPV vaccination. P-values and significance were calculated with a linear mixed model fitting each primary outcome with time, cohort, and their interaction as main effects. **b.** Violin plots of IgG concentrations are shown at W2, p-values were calculated with a T-test on log10-transformed values. IgG concentration values are shown on the log10 scale. Data are N = 16 – 25 per cohort. Sample means are plotted with 95% confidence intervals (solid black point and line), * p <0.05; ** p <0.01; *** p <0.001; ns, not significant. Abbreviations: pertussis toxin (PT), diphtheria toxoid (DIPH), tetanus toxin (TET), pertactin (PRN), filamentous haemagglutinin (FHA).

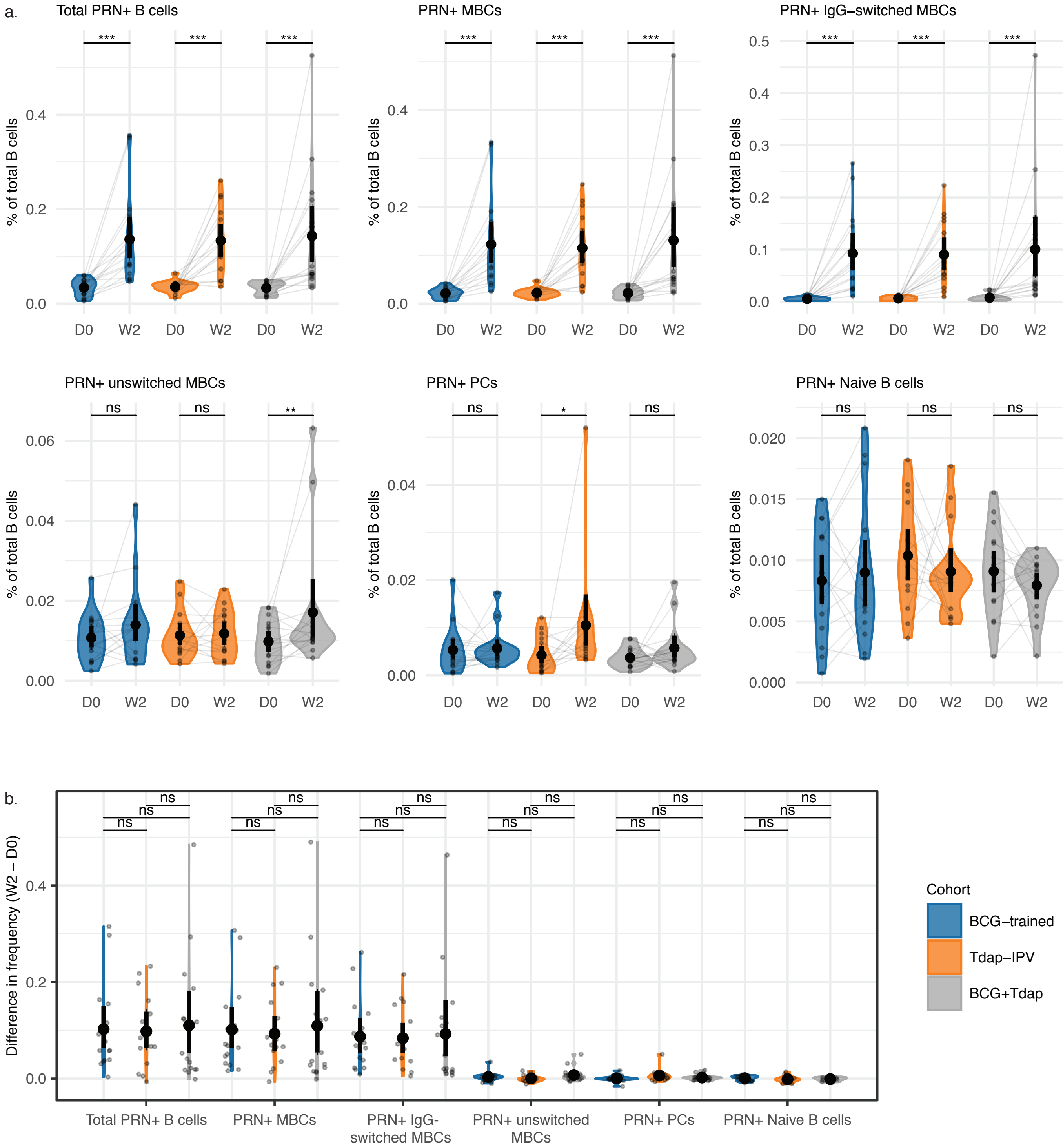

Supplementary Figure 3. Pre- and post- Tdap-IPV vaccination PRN+ B-cell responses. Violin plots are shown for **a.** frequencies of PRN+ B cell populations at baseline of Tdap-IPV vaccination (D0) and two weeks (W2) post immunization, and **b.** differences in frequency (W2 – D0) of the specified PRN+ B cell populations. Abbreviations: MBCs, memory B cells; PCs, plasma cells. Data are N = 15 – 16 per cohort. P-values and significance were calculated with a linear mixed model fitting each primary outcome with time, cohort, and their interaction as main effects. Sample means are plotted with 95% confidence intervals (solid black point and line), * p <0.05; ** p <0.01; *** p <0.001; ns, not significant.

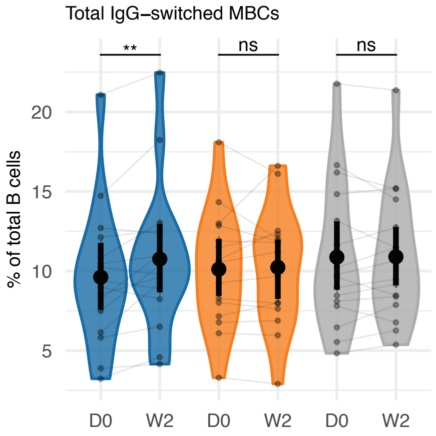

Supplementary Figure 4. Pre- and post- Tdap-IPV vaccination total IgG-switched memory B-cell frequencies. Violin plots of total IgG-switched memory B-cell (MBC) frequencies at baseline of Tdap-IPV vaccination (D0) and two weeks (W2) post immunization. Data are N = 15 – 16 per cohort. P-values and significance were calculated with a linear mixed model fitting each primary outcome with time, cohort, and their interaction as main effects. Sample means are plotted with 95% confidence intervals (solid black point and line), ** p <0.01; ns, not significant.

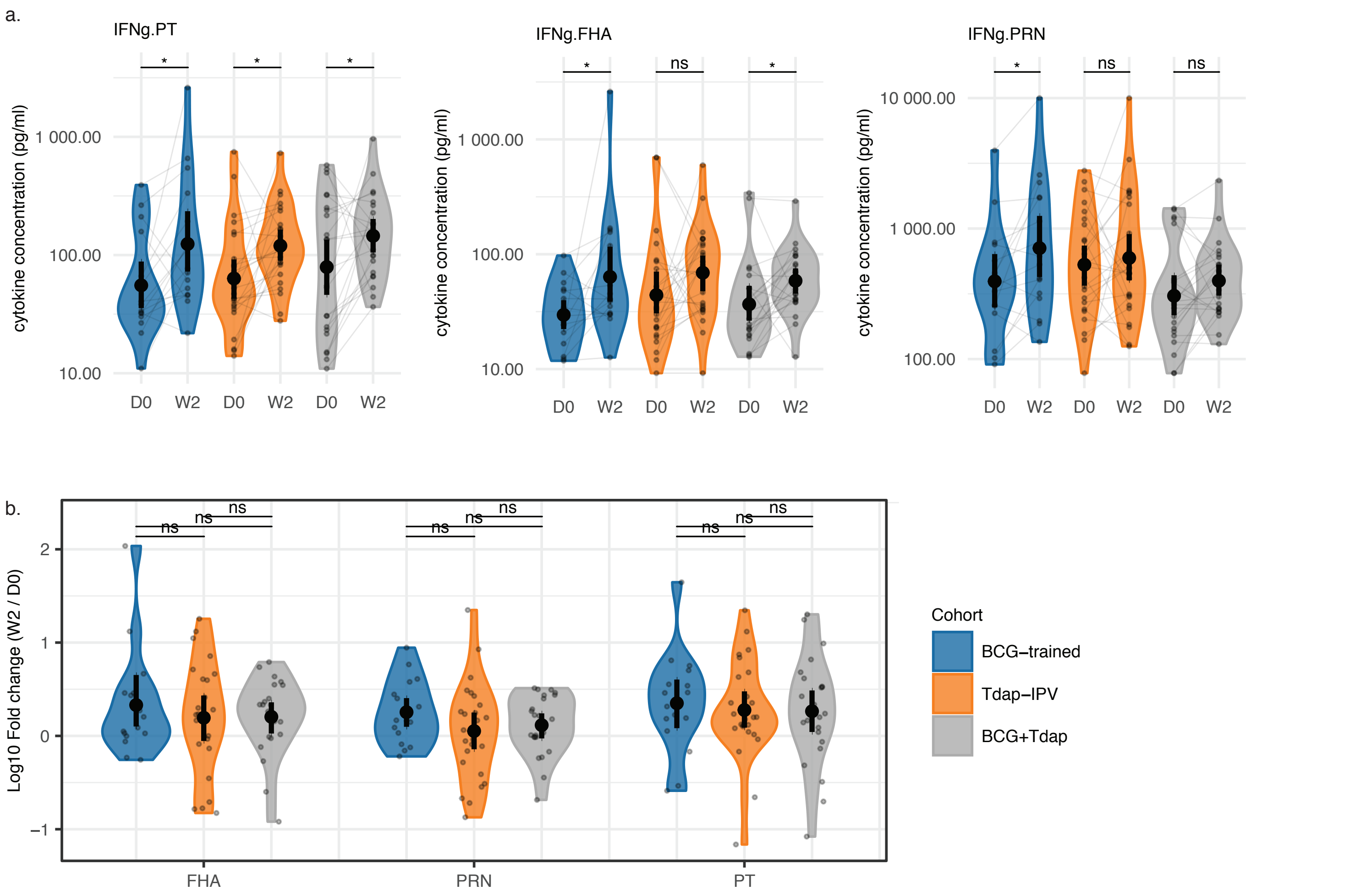

Supplementary Figure 5. Pre- and post- Tdap-IPV vaccination IFNg responses to stimulation with pertussis antigens. a. Violin plots of concentrations of IFNg in supernatant of peripheral blood mononuclear cells following PRN, FHA, or PT stimulation. Shown are measurements taken at baseline of Tdap-IPV vaccination (D0) and two weeks (W2) post immunization. Cytokine concentration values are shown on the log10 scale. **b.** Log10 fold change (W2 / D0) of cytokine responses of a. Data are N = 16 – 25 per cohort. P-values and significance were calculated with a linear mixed model fitting each primary outcome with time, cohort, and their interaction as main effects. Sample means are plotted with 95% confidence intervals (solid black point and line), * p <0.05; ns, not significant.

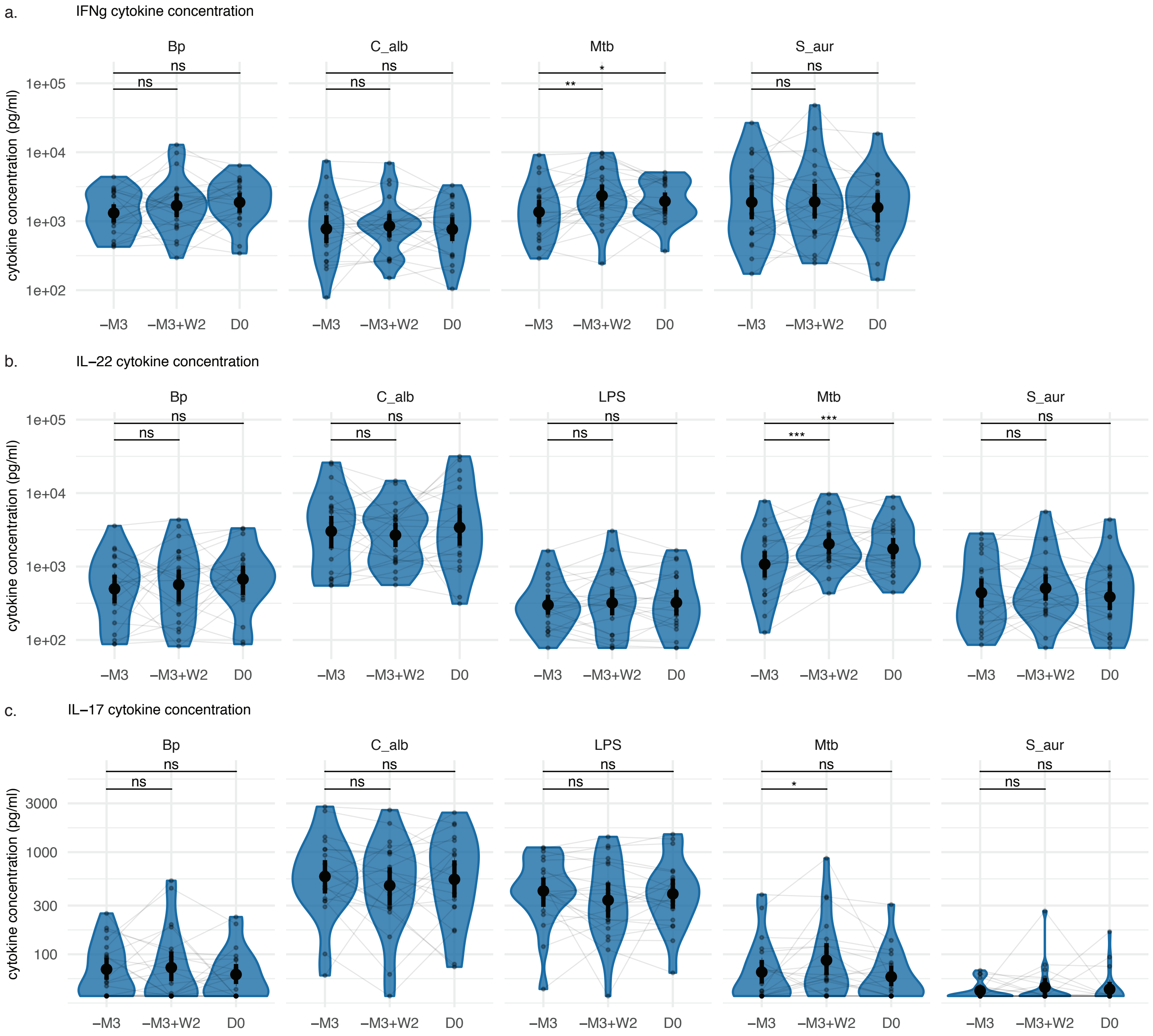

Supplementary Figure 6. Pre- and post- BCG vaccination heterologous IFNg, IL-22, and IL-17 cytokine concentrations. Violin plots of **a.** IFNg, **b.** IL-22, and **c.** IL-17 cytokine concentrations produced by peripheral blood mononuclear cells in response to LPS or heat-killed microorganisms (*Bordetella pertussis* (Bp), *Candida albicans* (C_alb), *Mycobacterium tuberculosis* (Mtb), *Staphylococcus aureus* (S_aur)) are shown. Cytokines were measured at the baseline of BCG vaccination (-M3), two weeks (-M3+W2) and three months after BCG vaccination (D0), which corresponds to the baseline of Tdap-IPV vaccination. Cytokine concentration values are shown on the log10 scale. Data are N = 20 - 23 subjects in the BCG-trained cohort. P-values and significance were calculated with a linear mixed model fitting each primary outcome with time, cohort, and their interaction as main effects. Sample means are plotted with 95% confidence intervals (solid black point and line), * $p < 0.05$; ** $p < 0.01$; ns, not significant.

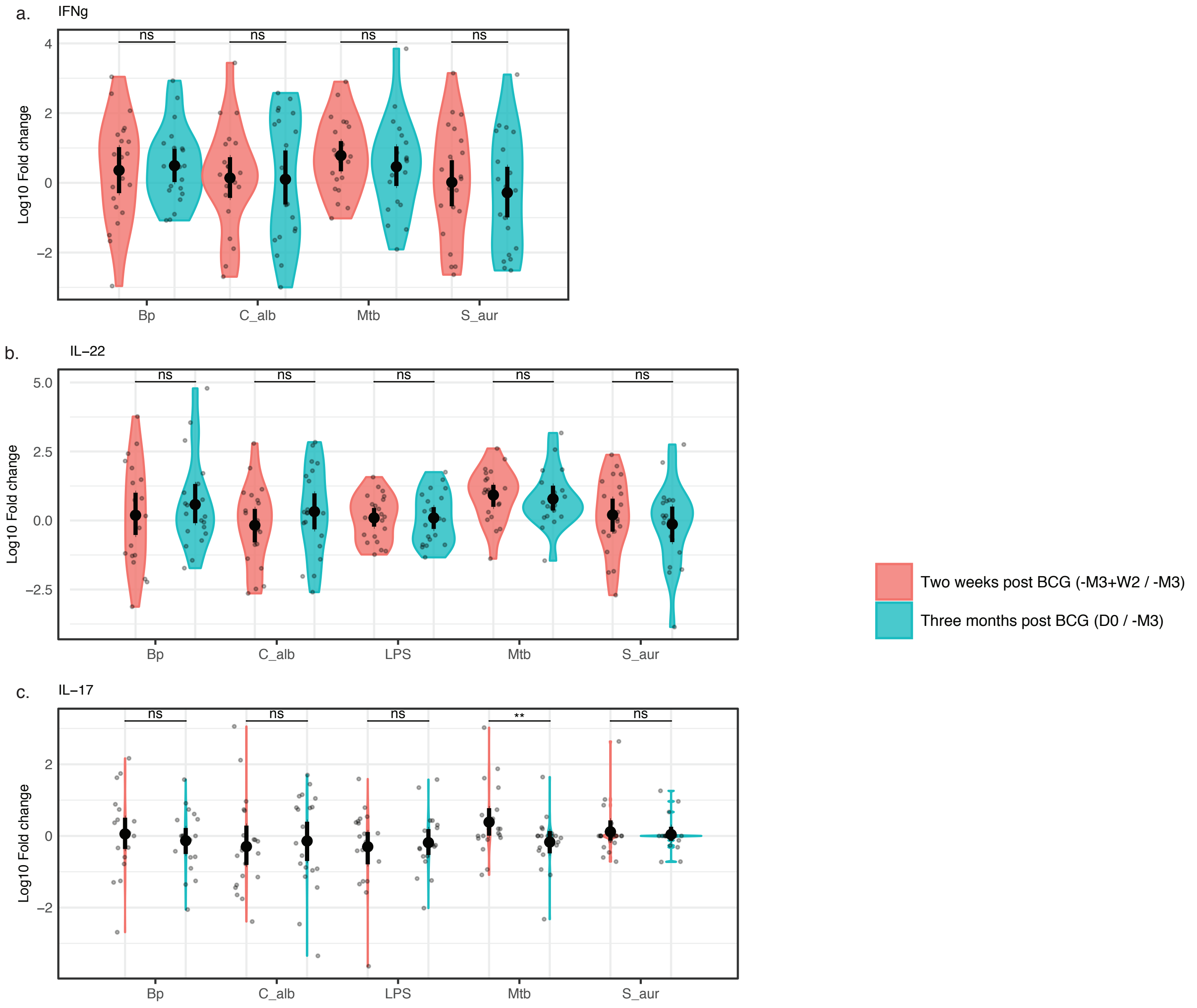

Supplementary Figure 7. Heterologous IFNg, IL-22, and IL-17 responses (related to Supplemental Figure 6). Violin plots for log10 fold change over baseline: **a.** IFNg, **b.** IL-22, and **c.** IL-17 cytokine responses two weeks and three months post-vaccination with BCG. Data are N = 22 - 23 subjects in the BCG-trained cohort. P-values and significance were calculated with a linear mixed model fitting each primary outcome with time, cohort, and their interaction as main effects. Sample means are plotted with 95% confidence intervals (solid black point and line), ** p <0.01; ns, not significant. Abbreviations: *Bordetella pertussis* (Bp), *Candida albicans* (C_alb), *Mycobacterium tuberculosis* (Mtb), *Staphylococcus aureus* (S_aur).

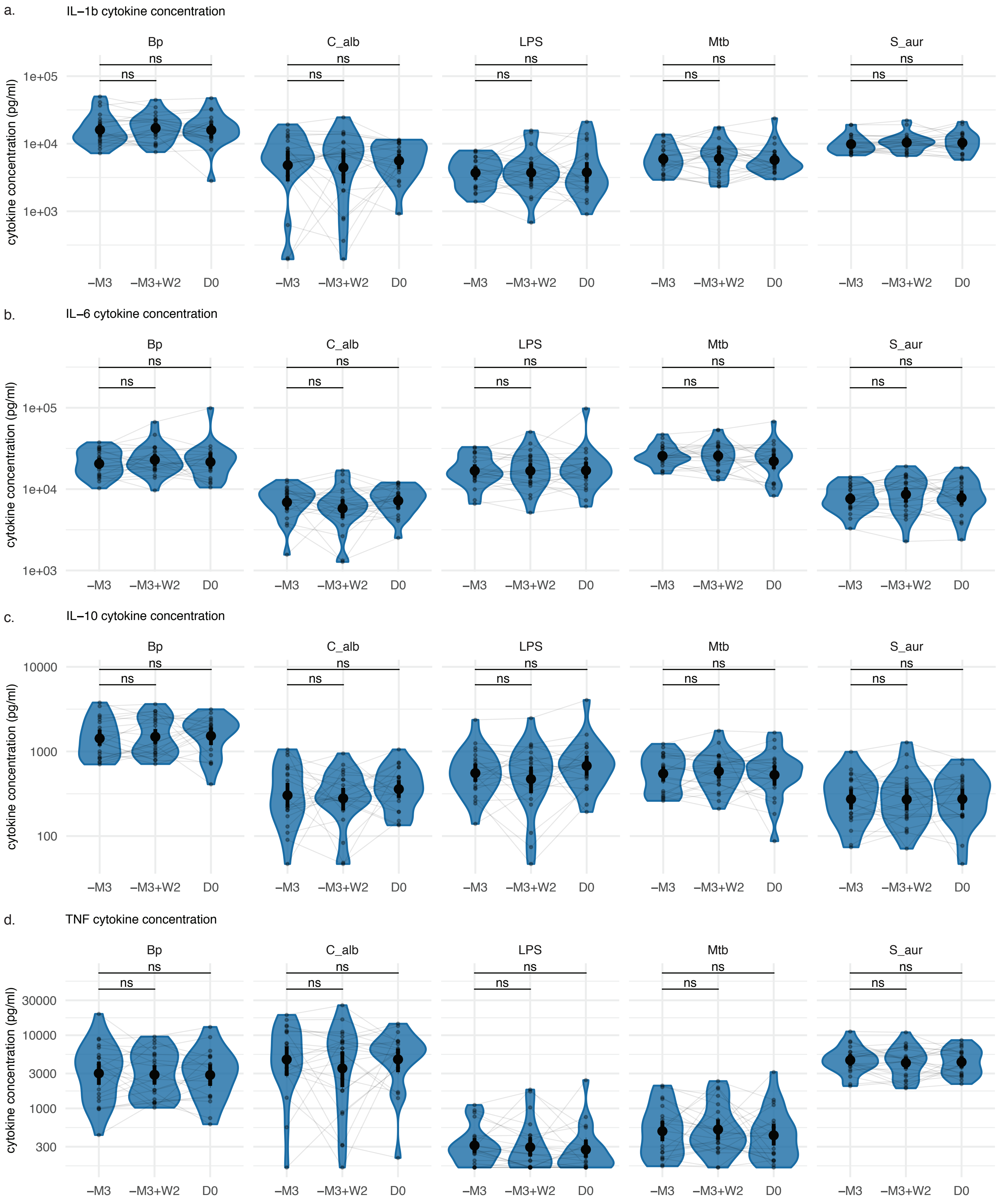

Supplementary Figure 8. Pre- and post- BCG vaccination trained immunity IL-1b, IL-6, IL-10, and TNF cytokine concentrations. Violin plots of **a.** IL-1b, **b.** IL-6, **c.** IL-10, and **d.** TNF cytokine concentrations produced by peripheral blood mononuclear cells in response to stimulation with lipopolysaccharide (LPS) or heat-killed microorganisms (*Bordetella pertussis* (Bp), *Candida albicans* (C_alb), *Mycobacterium tuberculosis* (Mtb), *Staphylococcus aureus* (S_aur)) are shown. Cytokines were measured at the baseline of BCG vaccination (-M3), two weeks (-M3+W2) and three months after BCG vaccination (D0), which corresponds to the baseline of Tdap-IPV vaccination. Cytokine concentration values are shown on the log10 scale. Data are N = 20 - 23 subjects in the BCG-trained cohort. P-values and significance were calculated with a linear mixed model fitting each primary outcome with time, cohort, and their interaction as main effects. Sample means are plotted with 95% confidence intervals (solid black point and line), ** p <0.01; ns, not significant.

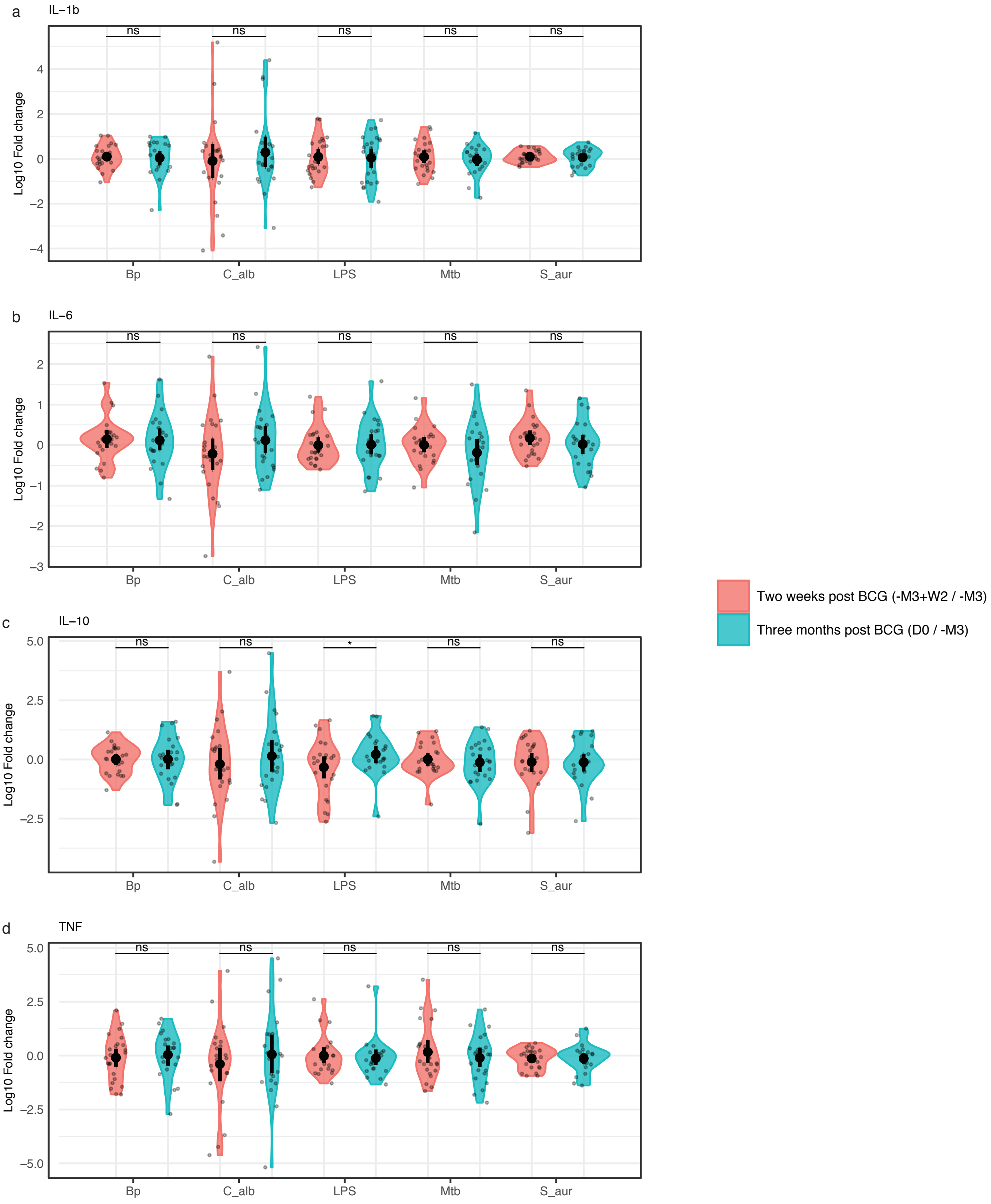

Supplementary Figure 9. Trained immunity cytokine responses IL-1b, IL-6, and IL-10, and TNF (related to Supplemental Figure 8). Violin plots for log10 fold change over baseline: **a.** IL-1b, **b.** IL-6, and **c.** IL-10, and **d.** TNF cytokine responses two weeks and three months post-vaccination with BCG. Data are N = 22 - 23 subjects in the BCG-trained cohort. P-values and significance were calculated with a linear mixed model fitting each primary outcome with time, cohort, and their interaction as main effects. Sample means are plotted with 95% confidence intervals (solid black point and line), * $p < 0.05$; ns, not signficant. Abbreviations: *Bordetella pertussis* (Bp), *Candida albicans* (C_alb), *Mycobacterium tuberculosis* (Mtb), *Staphylococcus aureus* (S_aur).

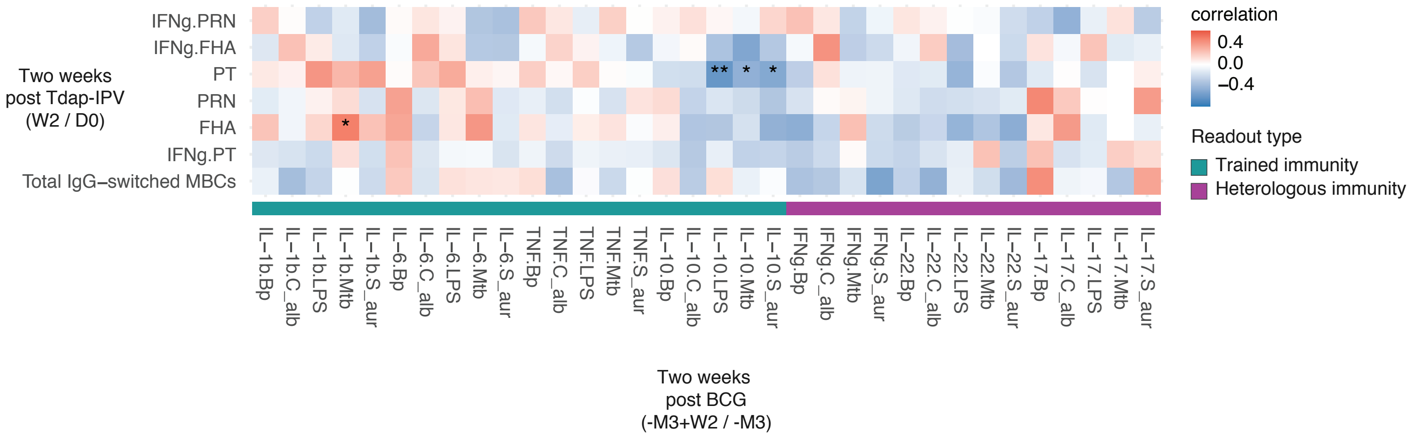

Supplementary Figure 10. Increases in cytokines two weeks following BCG vaccination are not associated with increases in pertussis-specific antibody, IFNg, and total IgG-switched MBC responses following Tdap-IPV. Within the BCG-trained cohort, increases for the indicated cytokine:stimulation combinations were calculated at two weeks post BCG (-M3+W2 / -M3). Pearson correlations between the cytokine variables on the x-axis and primary outcome responses on the y-axis: pertussis toxin (PT), pertactin (PRN), filamentous haemagglutinin (FHA) IgG, pertussis-specific IFNg responses (IFNg.PT, IFNg.PRN, IFNg.FHA), and total IgG-switched memory B cells (MBCs) two weeks post Tdap-IPV vaccination (W2 – D0), * $p < 0.05$; ** $p < 0.01$.

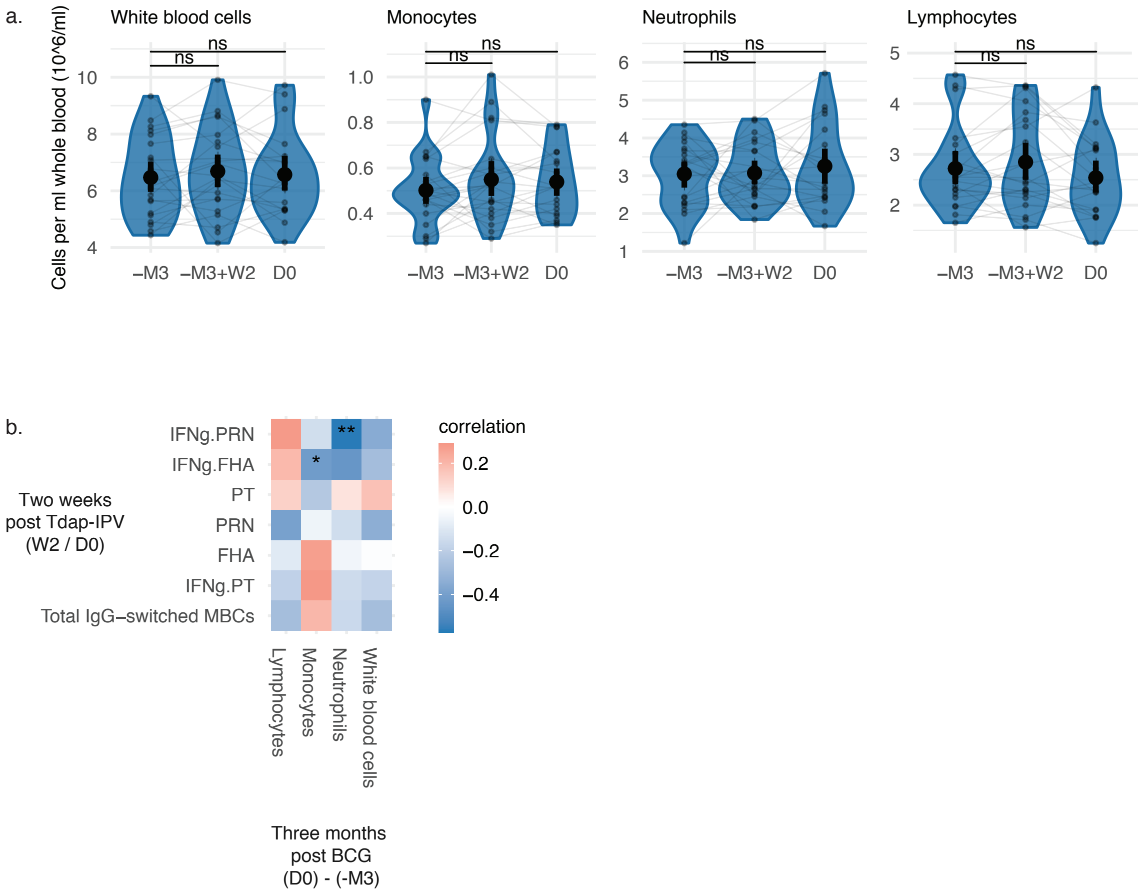

Supplementary Figure 11. Peripheral blood cell numbers and correlations with pertussis endpoints. a. Violin plots of cell numbers for the indicated cell types are shown at the baseline of BCG vaccination (-M3), two weeks (-M3+W2) and three months after BCG vaccination (D0), which corresponds to the baseline of Tdap-IPV vaccination. Data are N = 21 subjects in the BCG-trained cohort. P-values and significance were calculated with a linear mixed model fitting each primary outcome with time, cohort, and their interaction as main effects. Sample means are plotted with 95% confidence intervals (solid black point and line). **b.** Changes in abundance were calculated at three weeks post BCG (D0 / -M3) and Pearson correlations were calculated between changes in abundance on the x-axis and pertussis primary outcome responses two weeks post Tdap-IPV vaccination on the y-axis: Log10 fold change (W2 / D0) pertussis toxin (PT), pertactin (PRN), filamentous haemagglutinin (FHA) IgG, pertussis-specific IFNg responses (IFNg.PT, IFNg.PRN, IFNg.FHA), and total IgG-switched memory B cells (MBCs, W2 – D0). * p <0.05; ** p <0.01; ns, not significant. Data are N = 16 subjects in the BCG-trained cohort.

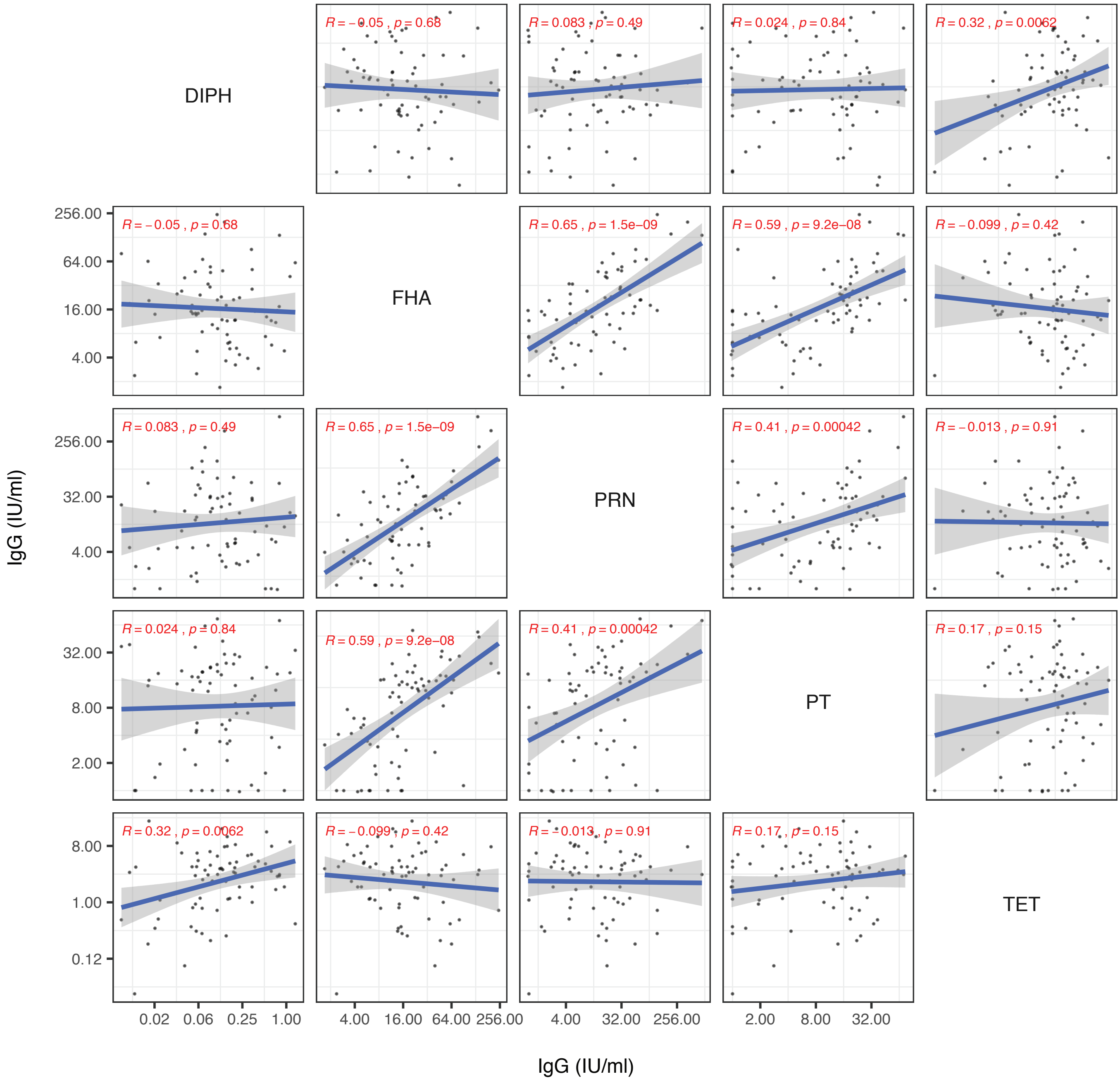

Supplementary Figure 12. Pertussis-antigen specific baseline antibody concentrations are highly correlated. Scatterplot matrix representing Pearson correlations between log2-transformed IgG concentrations for the Tdap-IPV antigens. Correlation coefficients and associated p-values are shown in red, and a regression line with 95% confidence intervals is also depicted. Data are N = 70 subjects, antibody concentration values are shown on the log2 scale and are derived from the baseline of Tdap-IPV vaccination (D0). Abbreviations: pertussis toxin (PT), diphtheria toxoid (DIPH), tetanus toxin (TET), pertactin (PRN), filamentous haemagglutinin (FHA).

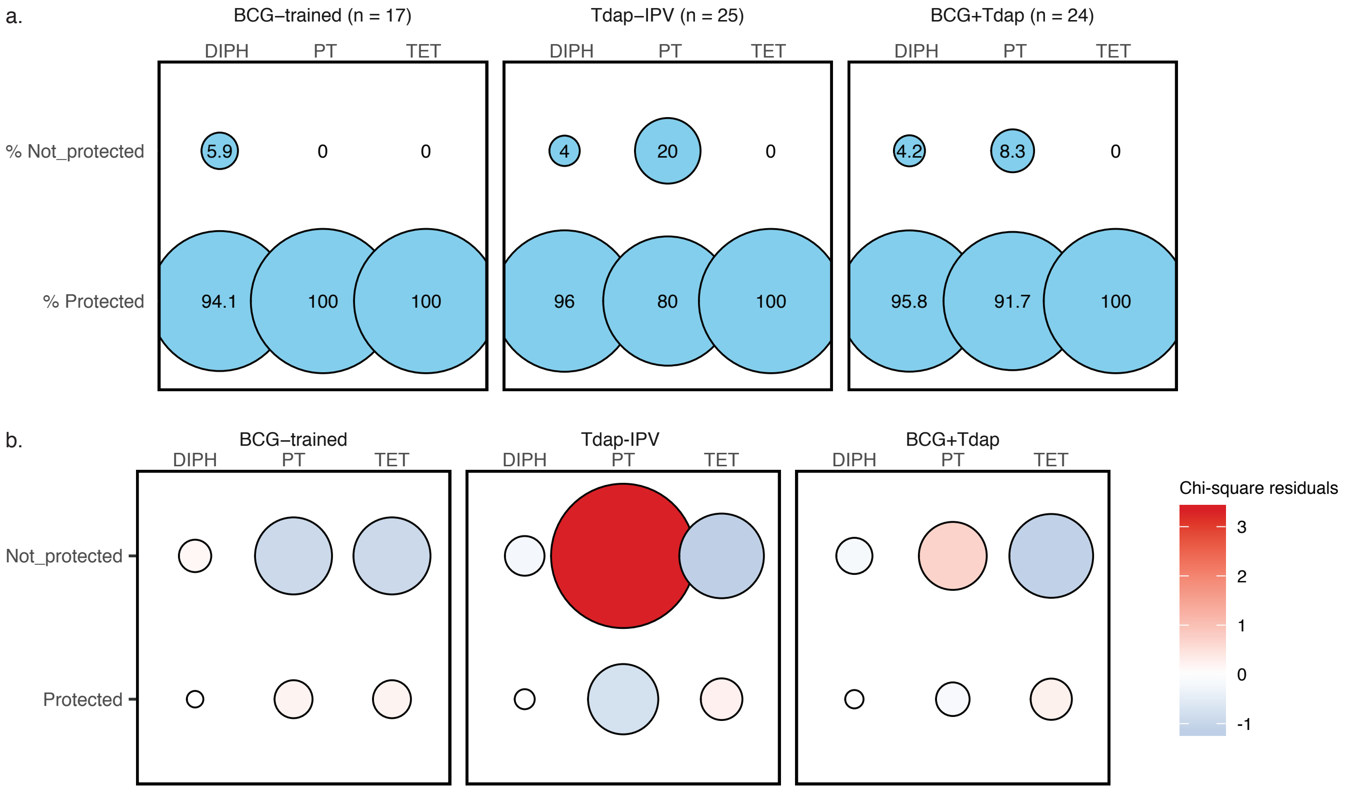

Supplementary Figure 13. BCG immunization improves pertussis toxin seroprotection rates following Tdap-IPV immunization. a. Seroprotection rates for pertussis toxin (PT), diphtheria toxoid (DIPH), and tetanus toxoid (TET), across the three study cohorts. **b.** A Fisher exact test was performed (P = 0.04) to assess the relationship between seroprotection status (Not protected vs protected) for each study cohort and for each vaccine antigen. In order to examine the association with seroprotection, a chi-square goodness-of-fit test was performed. Residuals are plotted to highlight important positive (red) and negative (blue) associations.

Supplementary Figure 14. CONSORT 2010 checklist (pg. 1/2)

CONSORT 2010 checklist of information to include when reporting a randomised trial*

Section/Topic	Item No	Checklist item	Reported on page No
**Title and abstract**			
	1a	Identification as a randomised trial in the title	1
	1b	Structured summary of trial design, methods, results, and conclusions (for specific guidance see CONSORT for abstracts)	2
**Introduction**			
Background and objectives	2a	Scientific background and explanation of rationale	3-4
	2b	Specific objectives or hypotheses	4
**Methods**			
Trial design	3a	Description of trial design (such as parallel, factorial) including allocation ratio	5
	3b	Important changes to methods after trial commencement (such as eligibility criteria), with reasons	NA
Participants	4a	Eligibility criteria for participants	14-15
	4b	Settings and locations where the data were collected	14-15
Interventions	5	The interventions for each group with sufficient details to allow replication, including how and when they were actually administered	5, 14-15
Outcomes	6a	Completely defined pre-specified primary and secondary outcome measures, including how and when they were assessed	5-6
	6b	Any changes to trial outcomes after the trial commenced, with reasons	NA
Sample size	7a	How sample size was determined	14
	7b	When applicable, explanation of any interim analyses and stopping guidelines	NA
Randomisation:			
Sequence generation	8a	Method used to generate the random allocation sequence	14
	8b	Type of randomisation; details of any restriction (such as blocking and block size)	NA
Allocation concealment mechanism	9	Mechanism used to implement the random allocation sequence (such as sequentially numbered containers), describing any steps taken to conceal the sequence until interventions were assigned	15
Implementation	10	Who generated the random allocation sequence, who enrolled participants, and who assigned participants to interventions	NA
Blinding	11a	If done, who was blinded after assignment to interventions (for example, participants, care providers, those	NA

Supplementary Figure 14. CONSORT 2010 checklist (pg. 2/2)

		assessing outcomes) and how	
	11b	If relevant, description of the similarity of interventions	NA
Statistical methods	12a	Statistical methods used to compare groups for primary and secondary outcomes	17-18
	12b	Methods for additional analyses, such as subgroup analyses and adjusted analyses	17-18
**Results**			
Participant flow (a diagram is strongly recommended)	13a	For each group, the numbers of participants who were randomly assigned, received intended treatment, and were analysed for the primary outcome	5
	13b	For each group, losses and exclusions after randomisation, together with reasons	5
Recruitment	14a	Dates defining the periods of recruitment and follow-up	14
	14b	Why the trial ended or was stopped	NA
Baseline data	15	A table showing baseline demographic and clinical characteristics for each group	5
Numbers analysed	16	For each group, number of participants (denominator) included in each analysis and whether the analysis was by original assigned groups	5
Outcomes and estimation	17a	For each primary and secondary outcome, results for each group, and the estimated effect size and its precision (such as 95% confidence interval)	6-9
	17b	For binary outcomes, presentation of both absolute and relative effect sizes is recommended	NA
Ancillary analyses	18	Results of any other analyses performed, including subgroup analyses and adjusted analyses, distinguishing pre-specified from exploratory	6-9
Harms	19	All important harms or unintended effects in each group (for specific guidance see CONSORT for harms)	NA
**Discussion**			
Limitations	20	Trial limitations, addressing sources of potential bias, imprecision, and, if relevant, multiplicity of analyses	10-14
Generalisability	21	Generalisability (external validity, applicability) of the trial findings	10-14
Interpretation	22	Interpretation consistent with results, balancing benefits and harms, and considering other relevant evidence	10-14
**Other information**			
Registration	23	Registration number and name of trial registry	14
Protocol	24	Where the full trial protocol can be accessed, if available	14
Funding	25	Sources of funding and other support (such as supply of drugs), role of funders	19

*We strongly recommend reading this statement in conjunction with the CONSORT 2010 Explanation and Elaboration for important clarifications on all the items. If relevant, we also recommend reading CONSORT extensions for cluster randomised trials, non-inferiority and equivalence trials, non-pharmacological treatments, herbal interventions, and pragmatic trials. Additional extensions are forthcoming: for those and for up to date references relevant to this checklist, see www.consort-statement.org.

Supplementary Table 1. Post-vaccination effects within each cohort (pg. 1/2)

Response	Contrast	Cohort	estimate	lower.CL	upper.CL	df	t.ratio	p.value	Threshold
PRN	W2 - D0	BCG-trained	1.721648538	2.003158446	1.440138629	122.7379	-12.106039	1.11E-22	***
PRN	Y1 - D0	BCG-trained	1.091392529	1.374933325	0.807851733	120.1669	-7.6209516	6.44E-12	***
PRN	W2 - D0	Tdap-IPV	1.301661921	1.531865467	1.071458376	115.6623	-11.199588	3.63E-20	***
PRN	Y1 - D0	Tdap-IPV	0.917912748	1.163067939	0.672757558	117.7797	-7.4147007	2.04E-11	***
PRN	W2 - D0	BCG+Tdap	1.166192898	1.401285023	0.931100773	115.6783	-9.8253384	6.22E-17	***
PRN	Y1 - D0	BCG+Tdap	0.884603581	1.119695706	0.649511456	115.6783	-7.452909	1.80E-11	***
TET	W2 - D0	BCG-trained	0.639620574	0.880683991	0.398557157	131.4912	-5.2487351	5.98E-07	***
TET	Y1 - D0	BCG-trained	0.234921956	0.480334413	-0.010490502	129.2315	-1.8939175	0.060471122	ns
TET	W2 - D0	Tdap-IPV	0.7105017	0.915312143	0.505691257	118.9346	-6.8691432	3.15E-10	***
TET	Y1 - D0	Tdap-IPV	0.378245171	0.592828194	0.163662148	126.3532	-3.488234	0.000669717	***
TET	W2 - D0	BCG+Tdap	0.266794954	0.475925645	0.057664263	119.0037	-2.5260782	0.012847967	*
TET	Y1 - D0	BCG+Tdap	-0.06387607	0.145254619	-0.273006764	119.0037	0.60479389	0.546467419	ns
DIPH	W2 - D0	BCG-trained	0.943418112	1.150013742	0.736822482	122.6464	-9.0393577	2.84E-15	***
DIPH	Y1 - D0	BCG-trained	0.303250601	0.511316768	0.095184435	120.0971	-2.8856693	0.004631911	**
DIPH	W2 - D0	Tdap-IPV	0.79683954	0.965738111	0.62794097	115.6492	-9.3446117	8.35E-16	***
DIPH	Y1 - D0	Tdap-IPV	0.375446667	0.555328924	0.19556441	117.7333	-4.1332818	6.73E-05	***
DIPH	W2 - D0	BCG+Tdap	0.755425524	0.927910907	0.58294014	115.665	-8.6747125	3.02E-14	***
DIPH	Y1 - D0	BCG+Tdap	0.269324105	0.441809489	0.096838721	115.665	-3.0927062	0.002486488	**
PT	W2 - D0	BCG-trained	1.624374445	1.876466559	1.372282331	123.4409	-12.754209	2.77E-24	***
PT	Y1 - D0	BCG-trained	0.717609986	0.971710037	0.463509935	120.7135	-5.5912319	1.43E-07	***
PT	W2 - D0	Tdap-IPV	1.055101738	1.261674149	0.848529326	115.7689	-10.116578	1.28E-17	***
PT	Y1 - D0	Tdap-IPV	0.679223898	0.899077502	0.459370294	118.1524	-6.117845	1.27E-08	***
PT	W2 - D0	BCG+Tdap	1.34469053	1.555648709	1.133732351	115.7871	-12.625159	1.66E-23	***
PT	Y1 - D0	BCG+Tdap	0.742418687	0.953376865	0.531460508	115.7871	-6.9704918	2.07E-10	***
FHA	W2 - D0	BCG-trained	1.603785977	1.85598428	1.351587674	129.4901	-12.581433	3.29E-24	***
FHA	Y1 - D0	BCG-trained	1.044892473	1.300954043	0.788830903	126.5292	-8.075119	4.55E-13	***
FHA	W2 - D0	Tdap-IPV	1.173750653	1.385367383	0.962133922	117.5325	-10.984205	9.62E-20	***
FHA	Y1 - D0	Tdap-IPV	0.801669126	1.024857437	0.578480815	123.2514	-7.1097932	8.27E-11	***
FHA	W2 - D0	BCG+Tdap	1.258294111	1.474387664	1.042200558	117.5824	-11.531379	4.83E-21	***
FHA	Y1 - D0	BCG+Tdap	0.920736366	1.136829919	0.704642813	117.5824	-8.4378999	9.63E-14	***
IFNg.PT	W2 - D0	BCG-trained	0.353846884	0.625880135	0.081813633	68.63953	-2.5951657	0.011552091	*
IFNg.PT	W2 - D0	Tdap-IPV	0.27698864	0.498631421	0.055345859	59.99738	-2.4997894	0.015179055	*
IFNg.PT	W2 - D0	BCG+Tdap	0.268005584	0.500236078	0.03577509	62.61841	-2.3064598	0.024406766	*
IFNg.FHA	W2 - D0	BCG-trained	0.28051788	0.53368184	0.02735392	69.451	-2.2102397	0.030385806	*
IFNg.FHA	W2 - D0	Tdap-IPV	0.194172535	0.401912145	-0.013567074	60.27855	-1.8694845	0.0664149	ns
IFNg.FHA	W2 - D0	BCG+Tdap	0.221287657	0.438463507	0.004111806	63.00372	-2.0361729	0.04594199	*
IFNg.PRN	W2 - D0	BCG-trained	0.24577662	0.45497095	0.036582289	65.99751	-2.3457098	0.02200531	*
IFNg.PRN	W2 - D0	Tdap-IPV	0.051861485	0.219322885	-0.115599915	59.4393	-0.6195968	0.537891212	ns
IFNg.PRN	W2 - D0	BCG+Tdap	0.082453981	0.258929102	-0.09402114	61.54302	-0.9341119	0.353896852	ns
IL-22.PT	W2 - D0	BCG-trained	0.206860745	0.547869514	-0.134148024	69.24245	-1.2100865	0.230360555	ns
IL-22.PT	W2 - D0	Tdap-IPV	0.410963525	0.681963272	0.139963778	58.84248	-3.0346234	0.003582516	**
IL-22.PT	W2 - D0	BCG+Tdap	0.268023251	0.552658087	-0.016611584	61.26194	-1.8827607	0.064483384	ns
IL-22.FHA	W2 - D0	BCG-trained	0.072587029	0.337259279	-0.192085221	71.70663	-0.5467506	0.586247522	ns
IL-22.FHA	W2 - D0	Tdap-IPV	0.037092643	0.253210731	-0.179025446	59.79163	-0.3433385	0.73254818	ns
IL-22.FHA	W2 - D0	BCG+Tdap	0.009554028	0.235027884	-0.215919827	62.54013	-0.0846881	0.932779885	ns
IL-22.PRN	W2 - D0	BCG-trained	0.396295707	0.641210884	0.151380529	66.6808	-3.2300128	0.0019231	**
IL-22.PRN	W2 - D0	Tdap-IPV	0.135437227	0.330829623	-0.05995517	58.57614	-1.3872101	0.170633479	ns
IL-22.PRN	W2 - D0	BCG+Tdap	0.147517036	0.350240312	-0.055206239	59.73892	-1.4557013	0.150710622	ns

Supplementary Table 1. Post-vaccination effects within each cohort (pg. 2/2)

Response	Contrast	Cohort	estimate	lower.CL	upper.CL	df	t.ratio	p.value	Threshold
IL-17.PT	W2 - D0	BCG-trained	-0.05157577	0.192294644	-0.295446188	67.40467	0.4220861	0.674305438	ns
IL-17.PT	W2 - D0	Tdap-IPV	0.236771541	0.433693207	0.039849876	59.68796	-2.4053449	0.019274192	*
IL-17.PT	W2 - D0	BCG+Tdap	0.097708873	0.304635487	-0.109217741	62.0938	-0.9438679	0.348895367	ns
IL-17.FHA	W2 - D0	BCG-trained	-0.08118497	0.113312887	-0.275682823	68.61174	0.83279033	0.407852526	ns
IL-17.FHA	W2 - D0	Tdap-IPV	-0.04657405	0.114544903	-0.207692999	59.16456	0.57838653	0.565198425	ns
IL-17.FHA	W2 - D0	BCG+Tdap	-0.02402946	0.148388881	-0.196447806	65.02115	0.27833381	0.78163935	ns
IL-17.PRN	W2 - D0	BCG-trained	-0.18714014	0.078692667	-0.452972953	70.74066	1.40377914	0.164759471	ns
IL-17.PRN	W2 - D0	Tdap-IPV	0.057099451	0.273883102	-0.159684201	58.94382	-0.5270601	0.600129975	ns
IL-17.PRN	W2 - D0	BCG+Tdap	0.023657509	0.252577549	-0.205262531	63.16763	-0.2065057	0.837060062	ns
PRN+ IgG-switched MBCs	W2 - D0	BCG-trained	0.083338786	0.12257632	0.044101252	56.56901	-4.2538504	7.97E-05	***
PRN+ IgG-switched MBCs	W2 - D0	Tdap-IPV	0.078881015	0.117341368	0.040420661	57.97901	-4.1054933	0.000127968	***
PRN+ IgG-switched MBCs	W2 - D0	BCG+Tdap	0.090237356	0.128715288	0.051759425	54.64728	-4.700511	1.80E-05	***
PRN+ unswitched MBCs	W2 - D0	BCG-trained	0.000567319	0.005289713	-0.004155076	52.98323	-0.2409595	0.810516566	ns
PRN+ unswitched MBCs	W2 - D0	Tdap-IPV	0.000484266	0.005133287	-0.004164754	54.69604	-0.2087779	0.835397184	ns
PRN+ unswitched MBCs	W2 - D0	BCG+Tdap	0.006817888	0.011427443	0.002208333	51.55475	-2.9685958	0.004530225	**
PRN+ Naive B cells	W2 - D0	BCG-trained	-1.18E-05	0.002619441	-0.00264311	55.40948	0.009012	0.99284193	ns
PRN+ Naive B cells	W2 - D0	Tdap-IPV	-0.00088111	0.001701458	-0.003463679	56.97973	0.68319866	0.497250018	ns
PRN+ Naive B cells	W2 - D0	BCG+Tdap	-0.00118565	0.001390317	-0.003761625	53.62847	0.92294111	0.360171834	ns
PRN+ PCs	W2 - D0	BCG-trained	0.00058483	0.004504251	-0.003334591	56.56901	-0.2988438	0.766154319	ns
PRN+ PCs	W2 - D0	Tdap-IPV	0.004987498	0.008829287	0.001145709	57.97901	-2.5986954	0.011846654	*
PRN+ PCs	W2 - D0	BCG+Tdap	0.002098935	0.00594248	-0.001744609	54.64728	-1.0945549	0.278512722	ns
PRN+ MBCs	W2 - D0	BCG-trained	0.092667252	0.13615142	0.049183083	56.42449	-4.268312	7.61E-05	***
PRN+ MBCs	W2 - D0	Tdap-IPV	0.089634927	0.132264618	0.047005236	57.85792	-4.2091129	9.05E-05	***
PRN+ MBCs	W2 - D0	BCG+Tdap	0.107189793	0.149822574	0.064557012	54.51868	-5.0396848	5.47E-06	***
Total PRN+ B cells	W2 - D0	BCG-trained	0.093177032	0.137548716	0.048805348	56.34534	-4.2060784	9.40E-05	***
Total PRN+ B cells	W2 - D0	Tdap-IPV	0.09397992	0.137483521	0.050476318	57.79118	-4.3246011	6.12E-05	***
Total PRN+ B cells	W2 - D0	BCG+Tdap	0.108102799	0.151600447	0.064605151	54.44848	-4.9816989	6.73E-06	***
Total unswitched MBCs	W2 - D0	BCG-trained	1.180564011	2.614253974	-0.253125952	44.36246	-1.6591603	0.104137948	ns
Total unswitched MBCs	W2 - D0	Tdap-IPV	0.251634802	1.681836795	-1.17856719	44.784	-0.3544157	0.72469085	ns
Total unswitched MBCs	W2 - D0	BCG+Tdap	0.571556056	1.96094788	-0.817835768	44.20014	-0.8289586	0.411582183	ns
Total Naive B cells	W2 - D0	BCG-trained	-0.54851228	1.040161294	-2.13718585	43.59871	0.69601542	0.490113082	ns
Total Naive B cells	W2 - D0	Tdap-IPV	0.035334081	1.62228916	-1.551620999	43.79018	-0.0448789	0.96440787	ns
Total Naive B cells	W2 - D0	BCG+Tdap	-0.03543747	1.503378002	-1.574252944	43.52921	0.0464262	0.9631829	ns
Total PCs	W2 - D0	BCG-trained	-0.51351738	0.160937306	-1.187972058	51.63344	1.52808215	0.13259557	ns
Total PCs	W2 - D0	Tdap-IPV	-0.4599948	0.192396068	-1.112385677	51.32909	1.41530853	0.163016906	ns
Total PCs	W2 - D0	BCG+Tdap	-0.41717625	0.226310174	-1.060662668	48.65188	1.30305596	0.198689265	ns
Total MBCs	W2 - D0	BCG-trained	2.756772617	4.652471867	0.861073367	43.91758	-2.9309526	0.005346086	**
Total MBCs	W2 - D0	Tdap-IPV	0.335324661	2.22789654	-1.557247219	44.20707	-0.3570343	0.722764894	ns
Total MBCs	W2 - D0	BCG+Tdap	0.559521356	2.39610784	-1.277065127	43.80988	-0.6140628	0.542349632	ns
Total B cells	W2 - D0	BCG-trained	0.047522643	0.597209808	-0.502164523	47.13181	-0.1739101	0.862680917	ns
Total B cells	W2 - D0	Tdap-IPV	-0.36127212	0.184530011	-0.907074249	48.24348	1.3306866	0.189547698	ns
Total B cells	W2 - D0	BCG+Tdap	0.019722246	0.553469483	-0.514024991	46.59788	-0.0743518	0.941048711	ns
Total IgG-switched MBCs	W2 - D0	BCG-trained	1.076028517	1.767469617	0.384587416	43.80467	-3.136733	0.003050702	**
Total IgG-switched MBCs	W2 - D0	Tdap-IPV	0.125580409	0.816019453	-0.564858635	44.05976	-0.3665508	0.715708558	ns
Total IgG-switched MBCs	W2 - D0	BCG+Tdap	0.042963967	0.712794771	-0.626866837	43.71058	-0.1292929	0.897718995	ns

Supplementary Table 2. Differential effects between cohorts (pg. 1/2)

Response	Contrast	estimate	df	t.ratio	p.value	Threshold
PRN	(D0 - W2 BCG-trained) - (D0 - W2 Tdap-IPV)	-0.419986616	119.873157	-2.286698037	0.023968294	*
PRN	(D0 - W2 BCG-trained) - (D0 - W2 BCG+Tdap)	-0.55545564	119.807923	-2.998622956	0.003298712	**
PRN	(D0 - Y1 BCG-trained) - (D0 - Y1 Tdap-IPV)	-0.173479781	119.1423	-0.916427823	0.361293801	ns
PRN	(D0 - Y1 BCG-trained) - (D0 - Y1 BCG+Tdap)	-0.206788948	118.325586	-1.111755055	0.268497277	ns
PRN	(D0 - W2 Tdap-IPV) - (D0 - W2 BCG+Tdap)	-0.135469023	115.670464	-0.815487924	0.416469104	ns
PRN	(D0 - Y1 Tdap-IPV) - (D0 - Y1 BCG+Tdap)	-0.033309167	116.769952	-0.194218703	0.846342118	ns
TET	(D0 - W2 BCG-trained) - (D0 - W2 Tdap-IPV)	0.070881126	126.166447	0.443450141	0.658199688	ns
TET	(D0 - W2 BCG-trained) - (D0 - W2 BCG+Tdap)	-0.37282562	126.068328	-2.311939304	0.022403044	*
TET	(D0 - Y1 BCG-trained) - (D0 - Y1 Tdap-IPV)	0.143323215	127.981963	0.869920056	0.385972746	ns
TET	(D0 - Y1 BCG-trained) - (D0 - Y1 BCG+Tdap)	-0.298798028	124.883891	-1.834091274	0.069021043	ns
TET	(D0 - W2 Tdap-IPV) - (D0 - W2 BCG+Tdap)	-0.443706746	118.96985	-3.001490816	0.003274301	**
TET	(D0 - Y1 Tdap-IPV) - (D0 - Y1 BCG+Tdap)	-0.442121244	122.745819	-2.920799845	0.004156092	**
DIPH	(D0 - W2 BCG-trained) - (D0 - W2 Tdap-IPV)	-0.146578572	119.814545	-1.087586938	0.278960496	ns
DIPH	(D0 - W2 BCG-trained) - (D0 - W2 BCG+Tdap)	-0.187992588	119.749998	-1.383038795	0.169226304	ns
DIPH	(D0 - Y1 BCG-trained) - (D0 - Y1 Tdap-IPV)	0.072196066	119.082642	0.519751546	0.604201561	ns
DIPH	(D0 - Y1 BCG-trained) - (D0 - Y1 BCG+Tdap)	-0.033926496	118.27942	-0.248579791	0.804116937	ns
DIPH	(D0 - W2 Tdap-IPV) - (D0 - W2 BCG+Tdap)	-0.041414017	115.657286	-0.339790568	0.734630536	ns
DIPH	(D0 - Y1 Tdap-IPV) - (D0 - Y1 BCG+Tdap)	-0.106122562	116.739568	-0.843343992	0.400760786	ns
PT	(D0 - W2 BCG-trained) - (D0 - W2 Tdap-IPV)	-0.569272707	120.325181	-3.458222948	0.000752559	***
PT	(D0 - W2 BCG-trained) - (D0 - W2 BCG+Tdap)	-0.279683915	120.25478	-1.684578155	0.094663687	ns
PT	(D0 - Y1 BCG-trained) - (D0 - Y1 Tdap-IPV)	-0.038386088	119.61318	-0.226196964	0.821434307	ns
PT	(D0 - Y1 BCG-trained) - (D0 - Y1 BCG+Tdap)	0.024808701	118.688365	0.148747986	0.882004932	ns
PT	(D0 - W2 Tdap-IPV) - (D0 - W2 BCG+Tdap)	0.289588792	115.778183	1.942655254	0.054486007	ns
PT	(D0 - Y1 Tdap-IPV) - (D0 - Y1 BCG+Tdap)	0.063194789	117.014687	0.410751568	0.682005907	ns
FHA	(D0 - W2 BCG-trained) - (D0 - W2 Tdap-IPV)	-0.430035325	124.486363	-2.585332007	0.010880718	*
FHA	(D0 - W2 BCG-trained) - (D0 - W2 BCG+Tdap)	-0.345491866	124.3857	-2.058973414	0.041584116	*
FHA	(D0 - Y1 BCG-trained) - (D0 - Y1 Tdap-IPV)	-0.243223347	125.109798	-1.417127189	0.158931199	ns
FHA	(D0 - Y1 BCG-trained) - (D0 - Y1 BCG+Tdap)	-0.124156107	122.767946	-0.73350377	0.464649633	ns
FHA	(D0 - W2 Tdap-IPV) - (D0 - W2 BCG+Tdap)	0.084543458	117.557976	0.553558273	0.580932501	ns
FHA	(D0 - Y1 Tdap-IPV) - (D0 - Y1 BCG+Tdap)	0.11906724	120.489814	0.758824013	0.449439706	ns
IFNg.PT	(D0 - W2 BCG-trained) - (D0 - W2 Tdap-IPV)	-0.076858244	65.0556971	-0.43745354	0.663231313	ns
IFNg.PT	(D0 - W2 BCG-trained) - (D0 - W2 BCG+Tdap)	-0.0858413	66.0370111	-0.479172876	0.633398646	ns
IFNg.PT	(D0 - W2 Tdap-IPV) - (D0 - W2 BCG+Tdap)	-0.008983056	61.3547996	-0.055948152	0.95556491	ns
IFNg.FHA	(D0 - W2 BCG-trained) - (D0 - W2 Tdap-IPV)	-0.086345345	65.6103465	-0.52649795	0.60031772	ns
IFNg.FHA	(D0 - W2 BCG-trained) - (D0 - W2 BCG+Tdap)	-0.059230223	66.6459249	-0.354481496	0.724097262	ns
IFNg.FHA	(D0 - W2 Tdap-IPV) - (D0 - W2 BCG+Tdap)	0.027115121	61.6865129	0.18037217	0.857451663	ns
IFNg.PRN	(D0 - W2 BCG-trained) - (D0 - W2 Tdap-IPV)	-0.193915134	63.3533093	-1.445990472	0.153109065	ns
IFNg.PRN	(D0 - W2 BCG-trained) - (D0 - W2 BCG+Tdap)	-0.163322639	64.1076686	-1.192110184	0.237612258	ns
IFNg.PRN	(D0 - W2 Tdap-IPV) - (D0 - W2 BCG+Tdap)	0.030592496	60.5367265	0.251489141	0.802287726	ns
IL-22.PT	(D0 - W2 BCG-trained) - (D0 - W2 Tdap-IPV)	0.20410278	65.021891	0.935869329	0.352803408	ns
IL-22.PT	(D0 - W2 BCG-trained) - (D0 - W2 BCG+Tdap)	0.061162506	65.8532475	0.274937577	0.784224955	ns
IL-22.PT	(D0 - W2 Tdap-IPV) - (D0 - W2 BCG+Tdap)	-0.142940273	60.0989724	-0.727497106	0.469746199	ns
IL-22.FHA	(D0 - W2 BCG-trained) - (D0 - W2 Tdap-IPV)	-0.035494386	66.700784	-0.207370936	0.836351993	ns
IL-22.FHA	(D0 - W2 BCG-trained) - (D0 - W2 BCG+Tdap)	-0.063033001	67.7148616	-0.361801884	0.718626664	ns
IL-22.FHA	(D0 - W2 Tdap-IPV) - (D0 - W2 BCG+Tdap)	-0.027538615	61.2086859	-0.1763027	0.86063817	ns
IL-22.PRN	(D0 - W2 BCG-trained) - (D0 - W2 Tdap-IPV)	-0.26085848	63.4046781	-1.663666136	0.101114621	ns
IL-22.PRN	(D0 - W2 BCG-trained) - (D0 - W2 BCG+Tdap)	-0.24877867	63.7678685	-1.563362298	0.122916937	ns
IL-22.PRN	(D0 - W2 Tdap-IPV) - (D0 - W2 BCG+Tdap)	0.01207981	59.1759523	0.085844341	0.931879954	ns

Supplementary Table 2. Differential effects between cohorts (pg. 2/2)

Response	Contrast	estimate	df	t.ratio	p.value	Threshold
IL-17.PT	(D0 - W2 BCG-trained) - (D0 - W2 Tdap-IPV)	0.288347313	64.2475615	1.837666745	0.070736145	ns
IL-17.PT	(D0 - W2 BCG-trained) - (D0 - W2 BCG+Tdap)	0.149284645	65.1297871	0.932167637	0.354692671	ns
IL-17.PT	(D0 - W2 Tdap-IPV) - (D0 - W2 BCG+Tdap)	-0.139062668	60.9382295	-0.97349307	0.334156849	ns
IL-17.FHA	(D0 - W2 BCG-trained) - (D0 - W2 Tdap-IPV)	0.03461092	64.6068204	0.273729884	0.785165438	ns
IL-17.FHA	(D0 - W2 BCG-trained) - (D0 - W2 BCG+Tdap)	0.057155505	67.0103684	0.438920114	0.662131433	ns
IL-17.FHA	(D0 - W2 Tdap-IPV) - (D0 - W2 BCG+Tdap)	0.022544585	62.2237328	0.190962851	0.84917642	ns
IL-17.PRN	(D0 - W2 BCG-trained) - (D0 - W2 Tdap-IPV)	0.244239594	65.7889991	1.421809804	0.159805041	ns
IL-17.PRN	(D0 - W2 BCG-trained) - (D0 - W2 BCG+Tdap)	0.210797652	67.4226908	1.199259132	0.234624937	ns
IL-17.PRN	(D0 - W2 Tdap-IPV) - (D0 - W2 BCG+Tdap)	-0.033441942	61.1344562	-0.212096452	0.832737347	ns
PRN+ IgG-switched MBCs	(D0 - W2 BCG-trained) - (D0 - W2 Tdap-IPV)	-0.004457771	57.2563864	-0.162452166	0.871521768	ns
PRN+ IgG-switched MBCs	(D0 - W2 BCG-trained) - (D0 - W2 BCG+Tdap)	0.006898571	55.6192432	0.251504745	0.802350847	ns
PRN+ IgG-switched MBCs	(D0 - W2 Tdap-IPV) - (D0 - W2 BCG+Tdap)	0.011356341	56.2907236	0.418118276	0.677451453	ns
PRN+ unswitched MBCs	(D0 - W2 BCG-trained) - (D0 - W2 Tdap-IPV)	-8.31E-05	53.8199079	-0.025128737	0.98004522	ns
PRN+ unswitched MBCs	(D0 - W2 BCG-trained) - (D0 - W2 BCG+Tdap)	0.006250569	52.2813163	1.900407271	0.06290008	ns
PRN+ unswitched MBCs	(D0 - W2 Tdap-IPV) - (D0 - W2 BCG+Tdap)	0.006333621	53.116313	1.940336995	0.057655158	ns
PRN+ Naive B cells	(D0 - W2 BCG-trained) - (D0 - W2 Tdap-IPV)	-0.000869276	56.1752665	-0.472280525	0.638556177	ns
PRN+ Naive B cells	(D0 - W2 BCG-trained) - (D0 - W2 BCG+Tdap)	-0.001173819	54.5309357	-0.638963759	0.525521437	ns
PRN+ Naive B cells	(D0 - W2 Tdap-IPV) - (D0 - W2 BCG+Tdap)	-0.000304543	55.2853192	-0.167300843	0.867743798	ns
PRN+ PCs	(D0 - W2 BCG-trained) - (D0 - W2 Tdap-IPV)	0.004402668	57.2563869	1.606214457	0.113728494	ns
PRN+ PCs	(D0 - W2 BCG-trained) - (D0 - W2 BCG+Tdap)	0.001514105	55.6192437	0.552615394	0.582740987	ns
PRN+ PCs	(D0 - W2 Tdap-IPV) - (D0 - W2 BCG+Tdap)	-0.002888563	56.290724	-1.064687899	0.291562975	ns
PRN+ MBCs	(D0 - W2 BCG-trained) - (D0 - W2 Tdap-IPV)	-0.003032325	57.12331	-0.099710724	0.920923101	ns
PRN+ MBCs	(D0 - W2 BCG-trained) - (D0 - W2 BCG+Tdap)	0.014522541	55.482818	0.477828143	0.634649765	ns
PRN+ MBCs	(D0 - W2 Tdap-IPV) - (D0 - W2 BCG+Tdap)	0.017554866	56.1662826	0.5832624	0.56205147	ns
Total PRN+ B cells	(D0 - W2 BCG-trained) - (D0 - W2 Tdap-IPV)	0.000802888	57.0502257	0.025872562	0.979449274	ns
Total PRN+ B cells	(D0 - W2 BCG-trained) - (D0 - W2 BCG+Tdap)	0.014925767	55.4082146	0.481315498	0.632187727	ns
Total PRN+ B cells	(D0 - W2 Tdap-IPV) - (D0 - W2 BCG+Tdap)	0.01412288	56.0980394	0.459868636	0.647387911	ns
Total unswitched MBCs	(D0 - W2 BCG-trained) - (D0 - W2 Tdap-IPV)	-0.928929209	44.5721395	-0.924139671	0.360389847	ns
Total unswitched MBCs	(D0 - W2 BCG-trained) - (D0 - W2 BCG+Tdap)	-0.609007955	44.2837555	-0.614662524	0.541923153	ns
Total unswitched MBCs	(D0 - W2 Tdap-IPV) - (D0 - W2 BCG+Tdap)	0.319921254	44.499407	0.323253243	0.748017664	ns
Total Naive B cells	(D0 - W2 BCG-trained) - (D0 - W2 Tdap-IPV)	0.583846358	43.6942179	0.524111802	0.602849485	ns
Total Naive B cells	(D0 - W2 BCG-trained) - (D0 - W2 BCG+Tdap)	0.513074807	43.5650528	0.467650564	0.64236548	ns
Total Naive B cells	(D0 - W2 Tdap-IPV) - (D0 - W2 BCG+Tdap)	-0.070771552	43.6634804	-0.064537774	0.948836603	ns
Total PCs	(D0 - W2 BCG-trained) - (D0 - W2 Tdap-IPV)	0.053522572	51.4860886	0.114484248	0.909299234	ns
Total PCs	(D0 - W2 BCG-trained) - (D0 - W2 BCG+Tdap)	0.096341129	50.1898642	0.207567447	0.836406656	ns
Total PCs	(D0 - W2 Tdap-IPV) - (D0 - W2 BCG+Tdap)	0.042818557	49.990427	0.093856255	0.925598517	ns
Total MBCs	(D0 - W2 BCG-trained) - (D0 - W2 Tdap-IPV)	-2.421447956	44.0618058	-1.821738551	0.075287362	ns
Total MBCs	(D0 - W2 BCG-trained) - (D0 - W2 BCG+Tdap)	-2.19725126	43.8653937	-1.677866054	0.100485432	ns
Total MBCs	(D0 - W2 Tdap-IPV) - (D0 - W2 BCG+Tdap)	0.224196696	44.0139089	0.171330599	0.864749095	ns
Total B cells	(D0 - W2 BCG-trained) - (D0 - W2 Tdap-IPV)	-0.408794762	47.6801769	-1.061252136	0.293921465	ns
Total B cells	(D0 - W2 BCG-trained) - (D0 - W2 BCG+Tdap)	-0.027800396	46.8718449	-0.072999491	0.942117153	ns
Total B cells	(D0 - W2 Tdap-IPV) - (D0 - W2 BCG+Tdap)	0.380994365	47.4311975	1.003767684	0.320584149	ns
Total IgG-switched MBCs	(D0 - W2 BCG-trained) - (D0 - W2 Tdap-IPV)	-0.950448107	43.9318143	-1.960407088	0.056308853	ns
Total IgG-switched MBCs	(D0 - W2 BCG-trained) - (D0 - W2 BCG+Tdap)	-1.03306455	43.7590894	-2.163040572	0.036043157	*
Total IgG-switched MBCs	(D0 - W2 Tdap-IPV) - (D0 - W2 BCG+Tdap)	-0.082616442	43.8900555	-0.173097841	0.863370354	ns

Supplementary Table 3. B cell staining panel for flow cytometry

Fluorochrome	Target	Clone	Manufacturer	Catalog number
FITC	pertactin	NA	NA	NA
PE CF594	IgG	G18-145	BD Biosciences	562538
PECy7	CD19	J3-119	Beckman Coulter	IM3628
PerCPCy5.5	IgM	MHM-88	Biolegend	314512
APC	IgD	IA6-2	BD Biosciences	561303
APCH7	CD38	HB7	BD Biosciences	656646
OC515	CD45	GA90	Ctyognos	CYT-450C
CD27	BC421	O323	Biolegend	302824
FACS Buffer	NA	NA	NA	NA
