# Supplementary material for: BCG-induced trained immunity enhances acellular pertussis vaccination responses in an explorative randomized clinical trial
Source: NPJ Vaccines. 2022 Feb 17;7:21. doi: 10.1038/s41541-022-00438-4 (PMC8854388; doi:10.1038/s41541-022-00438-4)
Supplement: Supplementary file 2 — REPORTING SUMMARY [file 41541_2022_438_MOESM2_ESM.pdf]

nature portfolio

Corresponding author(s): Dr Dimitri Diavatopoulos

Last updated by author(s): 2021 Nov 23

Reporting Summary

Nature Portfolio wishes to improve the reproducibility of the work that we publish. This form provides structure for consistency and transparency in reporting. For further information on Nature Portfolio policies, see our Editorial Policies and the Editorial Policy Checklist.

Statistics

For all statistical analyses, confirm that the following items are present in the figure legend, table legend, main text, or Methods section.

n/a	Confirmed	
☐	☒	The exact sample size (*n*) for each experimental group/condition, given as a discrete number and unit of measurement
☐	☒	A statement on whether measurements were taken from distinct samples or whether the same sample was measured repeatedly
☐	☒	The statistical test(s) used AND whether they are one- or two-sided<br>*Only common tests should be described solely by name; describe more complex techniques in the Methods section.*
☐	☒	A description of all covariates tested
☐	☒	A description of any assumptions or corrections, such as tests of normality and adjustment for multiple comparisons
☐	☒	A full description of the statistical parameters including central tendency (e.g. means) or other basic estimates (e.g. regression coefficient) AND variation (e.g. standard deviation) or associated estimates of uncertainty (e.g. confidence intervals)
☐	☒	For null hypothesis testing, the test statistic (e.g. *F*, *t*, *r*) with confidence intervals, effect sizes, degrees of freedom and *P* value noted<br>*Give P values as exact values whenever suitable.*
☒	☐	For Bayesian analysis, information on the choice of priors and Markov chain Monte Carlo settings
☒	☐	For hierarchical and complex designs, identification of the appropriate level for tests and full reporting of outcomes
☐	☒	Estimates of effect sizes (e.g. Cohen's *d*, Pearson's *r*), indicating how they were calculated

Our web collection on statistics for biologists contains articles on many of the points above.

Software and code

Policy information about availability of computer code

Data collection	collection of flow cytometry data: LSRII flow cytometer (BD biosciences), antibody data: Bio-Plex LX200 (Bio-Rad), complete blood counts: Sysmex XN-450 haematology analyser
Data analysis	All statistical analyses were performed using the Rstudio environment (version 3.6.2), with libraries 'stats' (hypothesis tests and correlations), 'lme4', 'lmerTest' for mixed-effects modeling and associated p-values. Flow cytometry data analysis: Infinicyte software (Cytognios, Version 2.0).

For manuscripts utilizing custom algorithms or software that are central to the research but not yet described in published literature, software must be made available to editors and reviewers. We strongly encourage code deposition in a community repository (e.g. GitHub). See the Nature Portfolio guidelines for submitting code & software for further information.

Data

Policy information about availability of data

All manuscripts must include a data availability statement. This statement should provide the following information, where applicable:

- Accession codes, unique identifiers, or web links for publicly available datasets
- A description of any restrictions on data availability
- For clinical datasets or third party data, please ensure that the statement adheres to our policy

The data that support the findings of this study are available from the corresponding author upon reasonable request.

Field-specific reporting

Please select the one below that is the best fit for your research. If you are not sure, read the appropriate sections before making your selection.

☒ Life sciences ☐ Behavioural & social sciences ☐ Ecological, evolutionary & environmental sciences

For a reference copy of the document with all sections, see nature.com/documents/nr-reporting-summary-flat.pdf

Life sciences study design

All studies must disclose on these points even when the disclosure is negative.

Sample size	Seventy-five female volunteers were included. Cohort size was determined based on previous studies performed by our group in which we investigated the effects of BCG vaccination on innate immune responses.
Data exclusions	Two subjects were excluded from analysis for having a high anti-pertussis toxin IgG concentration prior to Tdap-IPV vaccination (>100IU/ml), indicative of recent infection with pertussis
Replication	Antibody measurements were made in duplicate.
Randomization	Randomization across the study arms was performed with a software-based algorithm.
Blinding	Experimental operators were blinded to treatment assignments.

Reporting for specific materials, systems and methods

We require information from authors about some types of materials, experimental systems and methods used in many studies. Here, indicate whether each material, system or method listed is relevant to your study. If you are not sure if a list item applies to your research, read the appropriate section before selecting a response.

Materials & experimental systems

n/a	Involved in the study	
☐	☒	Antibodies
☒	☐	Eukaryotic cell lines
☒	☐	Palaeontology and archaeology
☒	☐	Animals and other organisms
☐	☒	Human research participants
☐	☒	Clinical data
☒	☐	Dual use research of concern

Methods

n/a	Involved in the study	
☒	☐	ChIP-seq
☐	☒	Flow cytometry
☒	☐	MRI-based neuroimaging

Antibodies

Antibodies used	Flow cytometry antibodies: PE-CF594 Mouse Anti-Human IgG (BD biosciences cat. 562538), PE-Cy7 Mouse Anti-Human CD19 Monoclonal Antibody (IM3628 Beckman Coulter), PerCP/Cyanine5.5 Mouse anti-human IgM (314512, biolegend), APC Mouse Anti-Human IgD (BD Biosciences, cat. 561303), APC-H7 Mouse Anti-Human CD38 (BD Biosciences, cat. 656646), OC515 Mouse Anti-human CD45 (Cytognos CYT-450C), BV421 mouse anti-human CD27 (Biolegend 302824).<br><br>In house anti-human IgG for detection of PT-, PRN-, FHA-, TT- and DT- IgG antibodies.
Validation	All antibodies used are commercially available and validation statements are available from the respective manufacturer. Validation of in house anti-human IgG is provided in the publication: van Gageldonk et al. 2008 https://doi.org/10.1016/j.jim.2008.02.018

Human research participants

Policy information about studies involving human research participants

Population characteristics	The median age of the volunteers was 23 and all subjects are female.
Recruitment	Volunteers were recruited using bulletin boards and at Radboud University in Nijmegen and received moderate compensation.
Ethics oversight	The study was approved by the Arnhem-Nijmegen Medical Ethical Committee.

Note that full information on the approval of the study protocol must also be provided in the manuscript.

Clinical data

Policy information about clinical studies

All manuscripts should comply with the ICMJE guidelines for publication of clinical research and a completed CONSORT checklist must be included with all submissions.

Clinical trial registration	NCT02771782
Study protocol	The full trial protocol can be sent upon request to the corresponding author. The brief protocol is available at clinicaltrials.gov
Data collection	Data was collected at the Radboud University Medical Center (Nijmegen, the Netherlands) from March 2015 to July 2016
Outcomes	Primary outcomes were specified as increases in adaptive immune responses two weeks or one year after Tdap-IPV vaccination. We analyzed 31 immunological outcomes (antibody responses, PBMC re-stimulation with pertussis antigens, pertactin-specific B cell responses) in blood at day 0 and two weeks post Tdap-IPV immunisation. Antibody responses were also measured one year post-vaccination. We defined secondary outcomes for the BCG-trained cohort only as changes in cytokine production of PBMCs following stimulation with heat-killed Candida albicans (C_alb), Staphylococcus aureus (S_aur), Bordetella pertussis (Bp), or lipopolysaccharide (LPS). We quantified Th1/17 cytokines IFNg, IL-22, or IL-17 after 7 days as a readout for heterologous immunity, or monocyte-derived cytokines IL-10, IL-6, IL-1b, or TNF after 24 hours as a readout for trained immunity

Flow Cytometry

Plots

Confirm that:

- [x] The axis labels state the marker and fluorochrome used (e.g. CD4-FITC).
- [x] The axis scales are clearly visible. Include numbers along axes only for bottom left plot of group (a 'group' is an analysis of identical markers).
- [ ] All plots are contour plots with outliers or pseudocolor plots.
- [ ] A numerical value for number of cells or percentage (with statistics) is provided.

Methodology

Sample preparation	1-2 ml of freshly drawn whole blood collected in LiHep tubes was diluted in FACS buffer (PBS + 0.09% NaN3 + 0.2% bovine serum albumin, Calbiochem) and cells were pelleted and then washed twice with FACS buffer. Cells were resuspended in FACS buffer and then stained for 15 minutes with a B-cell phenotyping antibody panel, fixed (FACSLysing, BD Biosciences), and after another wash with FACS buffer were analysed within 1 hour of staining.
Instrument	LSRII (BD Biosciences)
Software	FACSDIva (BD bioscience) was used to collect the flow cytometry data. Infinicyte (v2.0) was used for analysis.
Cell population abundance	No cell sorting was not performed in this study.
Gating strategy	The boolean gating strategy is available in Supplementary figure 1. Starting from a raw FCS file, debris was excluded and CD45+ cells were identified. B cells were defined among CD45+ cells as CD19+. B cells were then stratified based on surface marker expression.

- [x] Tick this box to confirm that a figure exemplifying the gating strategy is provided in the Supplementary Information.
